# Electronic and optical properties of graphene, silicene, germanene, and their semi-hydrogenated systems†

**DOI:** 10.1039/d2ra06722f

**Published:** 2022-12-06

**Authors:** Vo Khuong Dien, Wei-Bang Li, Kuang-I. Lin, Nguyen Thi Han, Ming-Fa Lin

**Affiliations:** Department of Physics, National Cheng Kung University 701 Tainan Taiwan han.nguyen.dhsptn@gmail.com mflin@mail.ncku.edu.tw; Core Facility Center, National Cheng Kung University 701 Tainan Taiwan; Hierarchical Green-Energy Material (Hi-GEM) Research Center, National Cheng Kung University 701 Tainan Taiwan

## Abstract

We investigate the geometric, electric, and optical properties of two-dimensional honeycomb lattices using first-principles simulations. The main focus of this work is on the similarities and differences in their characteristics, as well as the delicate connection of orbital hybridizations and spin-polarizations with electronic and optical properties. Graphene, silicene, germanene, and their semi-hydrogenated systems, in turn, display sp^2^, sp^2^–sp^3^, and sp^3^s hybridizations. These bonding configurations are critical factors affecting the geometric structure, the electronic band structure, van Hove singularities in density of states, the magnetic configurations, the dielectric functions, and energy loss functions. Furthermore, the meta-stable and stable exciton states are expected to survive in pristine and semi-hydrogenated group IV monolayers, respectively. The theoretical predictions established in this work are important not only for basic science but also for high-tech applications.

## Abstract 2

We investigate the geometric, electric, and optical properties of two-dimensional honeycomb lattices using first-principles simulations.

## Graphical abstract

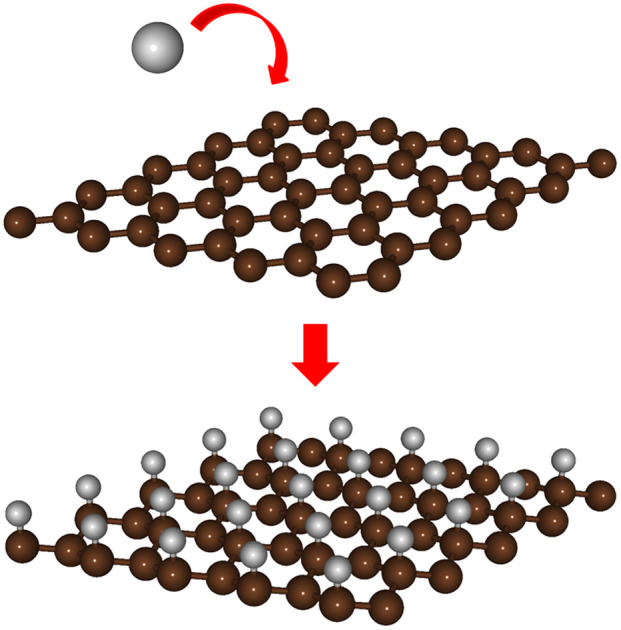

## 1 Introduction

Since monolayer graphene was successfully fabricated by mechanical exfoliation in 2004,^1^ emerging two-dimensional (2D) materials have attracted a great deal of experimental and theoretical attention.^2,3^ Beyond graphene, the other famous group-IV monolayers include silicene,^4^ germanene,^5^ tinene,^6^ and plumbene^7^ and it's hydrogenated systems.^8,9^ Such layered systems are ideal for studying a variety of physical, chemical, and material phenomena,^10–12^ mainly owing to rich and unique intrinsic atomic interactions and geometric symmetries.

Group-IV-related systems have so far been effectively synthesized by using various techniques, such as molecular beam epitaxial (MBE) growths^13^ and mechanical exfoliations.^14^ Graphene-like honeycomb lattices, such as 2D graphene, silicene, germanene, tinene, and plumbene, have been characterized on Cu(111),^15^ Ag(111),^16^ Al(111),^17^ Ag(111)^18^/InSb(111)^19^ and Pd_1−*x*_Pb_*x*_(111) alloy surfaces,^7^ respectively. Concerning hydrogenated systems, A. Geim's group developed a method for producing hydrogenated graphene by passing hydrogen gas through the electrical discharge.^7^ Wang *et al.*, report a new technique to fabricate high-quality graphane by plasma-enhanced chemical vapor deposition (CVD).^20^ The above experiments have proved that graphene gradually transforms from a zero-gap semimetal to a sizable band-gap semiconductor under hydrogen adsorption. Very interestingly, semi-hydrogenated graphene (graphone) also induces magnetism as a result of the breaking of symmetry.^20^

On the theoretical side, group-IV 2D pristine materials are predicted to display fascinating phenomena, including possible superconductivity,^21,22^ an observable quantum spin-Hall effect,^23^ and a significant electron–hole coupling.^24^ The current theoretical predictions showed that monolayer group-IV systems are gapless or direct-gap semiconductors in which the energy gap grows with the atomic number.^25^ In low-lying energy bands, graphene, silicene, and germanene are dominated by π bondings associated with p_*z*_ orbitals, while tinene and plumbene present considerable sp^3^-orbital hybridization.^25^ Such results are mainly controlled by the degree of buckling, the distribution ranges of the outer orbitals, and spin-orbit couplings (SOC). Absorption of hydrogen atoms is illustrated as an effective way to control the electronic and magnetic properties of group IV monolayers.^26–28^

The up-to-day research pointed out that the two-dimensional graphene, silicene, and germanene and related systems possess huge potential for many applications in nano-electronics,^29,30^ optoelectronics,^31^ spintronics,^30^ and energy storage fields.^32^ For example, the silicene field-effects transistor, which room-temperature mobility of approximately 100 cm^2^ V^−1^ s^−1^ was successfully fabricated, such invention pioneers for low-dimensional electronic devices.^33^ Graphene, silicene, and germanene have a distinct and wide absorbance spectrum, which suggests that they might be used for absorption in silicon technology, particularly in the terahertz regime.^34^ Because of the observed quantum spin Hall effects, silicene and germanene are also intriguing candidates for spintronic applications.^35^ The last but not least, group IV two-dimensional materials are considered promising in the field of electrochemical energy as well as hydrogen storage application due to their impressive properties such as ultrahigh surface-volume ratio, excellent mechanical strength, and flexibility.^36,37^

Despite numerous theoretical calculations and experimental measurements on group IV materials that have been conducted, certain fundamental knowledge is still not clarified. (i) The significant hybridizations related to the similarities and differences in characteristics of graphene, silicene, germanene, and their hydrogenated systems are not clear. (ii) Previous studies on the optical properties of group-IV-related systems have been done,^38,39^ but the critical mechanism, a close connection of initial and the final orbital hybridizations/spin-polarizations with the prominent optical excitations, is missing. (iii) Excitonic effects, the excited electron–hole interactions, are expected significant in low-dimensional materials. According to our knowledge, these coupled quasi-particles have been evaluated for group IV monolayers^24^ and their fully hydrogenated systems,^40,41^ but not for the semi-hydrogenated ones.

In this paper, the accurate simulation results and delicate analyses are capable of proposing significant pictures/mechanisms to thoroughly comprehend the geometric, electronic, magnetic properties, optical excitations, and excitonic effects of graphene, silicene, germanene, and related semi-hydrogenated systems: graphone, silicone, and germanone. The significant single-/multi-orbital hybridizations in various chemical bonds are obtained from the geometries, the electronic band structures, the spatial charge densities, and the charge density differences. Spin-split/spin-degenerate energy bands, spin density distributions, net magnetic moments, and spin-projected van Hove singularities are used to understand the magnetic configurations in hydrogenated systems. The energy-dependent optical excitations are thorough investigations of the dielectric function, absorption, reflectance coefficients, and energy loss function.^42–44^ Furthermore, the excitonic effects of group IV monolayers and related semi-hydrogenated systems are also considered in detail.

## 2 Computational details

### 2.1 Ground states calculations

To estimate the ground-state and exited-states characteristics of graphene-like related systems, first-principles calculations *via* the Vienna *Ab initio* Simulation Package (VASP)^39^ are utilized. We use the Perdew–Burke–Ernzerhof (PBE) approach in generalized gradient approximation (GGA)^45^ to estimate the exchange and correlation potential. The projected augmented wave (PAW) pseudopotentials^46^ are used to explain the electronic wave functions in the core region. The cutoff energy for the plane wave expansion is set to 500 eV. The Monkhorst–Pack sampling technique with a special *k*-point mesh of 30 × 30 × 1, and 200 × 200 × 1, respectively, is used for geometric optimization and electronic and optical calculations. The vacuum level is kept at about 20 Å to avoid interactions between the group IV monolayer with its image. The convergence condition of the ground state was set to 10^−8^ eV between two consecutive simulation steps, and all atoms are allowed to fully relax during the geometric optimization until the Hellmann–Feynman force^47^ acting on each atom was smaller than 0.01 eV Å^−1^.

### 2.2 Optical excitations

Under the perturbation of an electromagnetic wave, the electrons are vertically excited from the occupied states to the unoccupied ones in the energy spectrum. The interactions between photons and the carriers of the systems can be well characterized by the macroscopic dielectric functions *ε*(*ω*).^48^ This frequency-dependent function is very useful in understanding the main features of the energy loss functions, reflections, and absorption coefficients.

According to Fermi's golden rule,^49^ the picture for single-particle excitation/bare response function can be expressed by the imaginary part of the dielectric function:
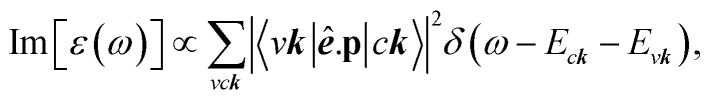
where the transition energy and the oscillation strength of each excitation peak are directly related to the joined density of state *δ*(*ω* − *E*_*c****k***_ − *E*_*v****k***_), and the square of the velocity matrix element, |〈*v****k***|***ê***.**p**|*c****k***〉|^2^, respectively. The bare response function mentioned here directly relates to the dynamic charge screening and reflects the main features of the electronic band structure.

In addition to the single-particle excitations, the energy loss functions/screen response function, being defined as 
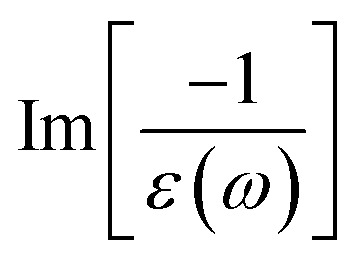
, could provide sufficient information about the collective excitations of an oscillation of valence electrons.^50^ Each prominence peak in the energy loss spectrum could be referred to as a plasmon mode – quantization of the coherent carrier oscillations at the long wave-length limit during the optical transitions. The imaginary part of dielectric functions Im[*ε*(*ω*)] (single-particle excitations), and energy loss functions 
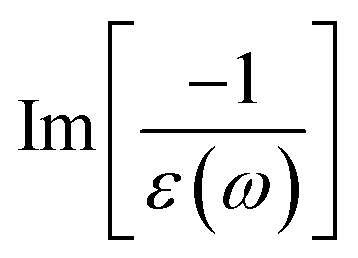
, (collective excitations) will dominate all optical phenomena in the condensed systems, *e.g.*, absorbance and reflectance coefficients. The close connection of the quasi-particle charges, spin-polarizations, orbital hybridizations, and optical excitations will be discussed in detail in the current work.

### 2.3 Many-body effects

The presence of exciton states, and excited electron–hole interactions, may have significant impacts on the optical responses.^48,51–54^ For example, causing the red-shift of the optical excitation peaks, modifying the absorbance spectrum, and creating new prominence structures below the fundamental band gap. Due to the absence of vertical charge screenings and the presence of quantum confinement effects, the excitonic effects in 2D materials are expected to be stronger than those of bulk counterparts.^55^ Of course, whether the stable exciton states could be survived or not also depend on other factors: the screening of other charges in materials and the kinetic of excited electrons/holes (effective mass). To evaluate the influence of excitonic effects on optical properties of group IV monolayers and related systems, we solve the standard Bethe–Salpeter equation (BSE)^56^ on the top of GW approximation wave functions^57^ (Details of computation please refer to ESI†).

## 3 Results and discussions

### 3.1 Graphene, silicene, and germanene

**Fig. 1:**
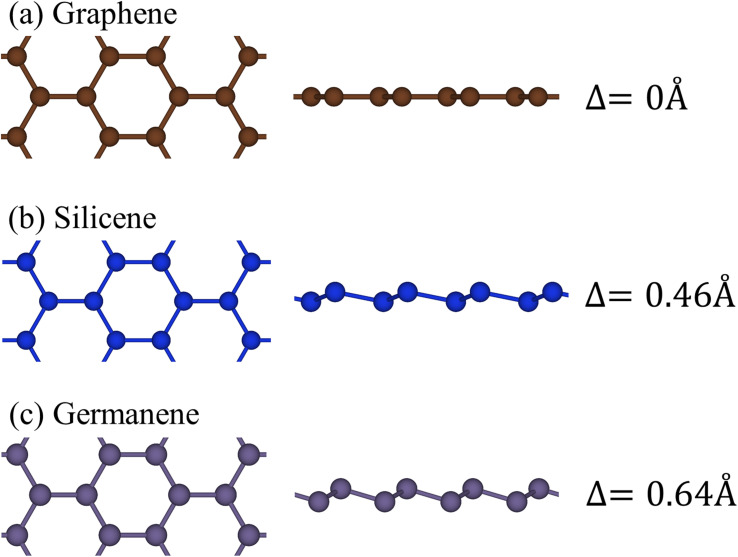
The side and top views of the geometric structure of (a) graphene, (b) silicene, and (c) germanene, respectively.

Group-IV monolayer materials exhibit diverse essential properties, mostly because of the hexagonal lattice, and the unique hybridizations.^25–27,62^ Generally, such materials could give full information about sp^2^ and sp^2^–sp^3^ hybridizations, which arise mainly due to the competition of π(p_*z*_) and σ(s, p_*x*_, p_*y*_) bondings. As for graphene, the significant hybridizations of 2s, 2p_*x*,_ and 2p_*y*_ orbitals of carbon atoms rise to the sp^2^ form. This hybridization leads to strong σ bonding between the two nearest atoms and thus keeps them in a plane. Furthermore, the π-bonding, which is due to the hybridization of p_*z*_ orbitals, extends to the vacuum and is well separated from the σ-bondings (see Fig. 1(a) and (b)). Go down to the silicene (Fig. 1(c) and (d)) and germanene (Fig. 1(e) and (f)), the chemical bonding between the two nearest atoms is not strong enough to keep the hexagon in the planar form, and thus, leads to the low-buckled structure. The height difference between the two sub-lattices (*Δ*) is equal to 0.46 Å and 0.64 Å for silicene and germanene, respectively. Due to this non-planar form, the p_*z*_ orbital partially merges with the (s, p_*x*_, p_*y*_) orbitals and thus creates sp^2^–sp^3^ hybridizations. The electronic and optical properties of graphene, silicene, and germanene are greatly influenced by these bonding features, as will be detailed below.

**Fig. 2:**
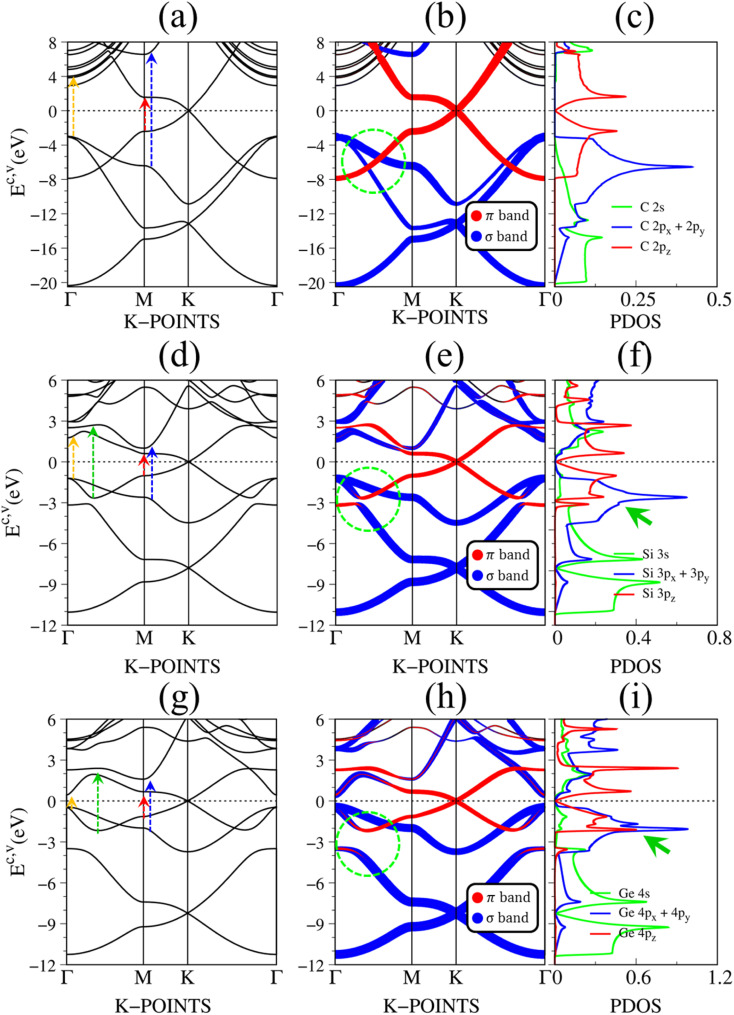
The DFT electronic band structure, the projected electronic wave functions, and the orbital-projected density of states of (a)–(c) graphene, (d)–(f) silicene, and (g)–(i) germanene, respectively. The colored arrows in (a), (d), and (g) indicates the optical excitations that correspond to the prominence absorption peaks in Fig. 3. The green circles in (b), (e), and (h) represent the crossing, and anti-crossing behaviors in graphene, silicene/germanene, respectively. While the green arrows in (f), and (i) point out the van Hove singularities that are related to the anti-crossing phenomena.

Fig. 2(a)–(c) depict the electronic band structure, the orbital decompound electronic band structure along the high-symmetry points, and the orbital projected density of states of group IV monolayers. As a consequence of the hexagonal symmetry, the electronic band structure of graphene is well characterized by the linear and isotropic Dirac cone.^2^ The π band goes along the ΓMK part with a bandwidth of about 7 eV. The graphene band structure also presents three energy sub-bands that belong to σ – (2p_*x*_, 2p_*y*_), and σ – 2s orbitals. The latter manifests at a very deep energy range (*E*_v_ < 6 eV) under the largest ionization energy.^25^ It is very important to note that the π and σ energy sub-bands are well separated (crossing behaviors), as demonstrated by the green circle in Fig. 2(b), which totally reflects the orthogonal of π and σ bondings. Furthermore, critical points, which are band-edge states with the vanishing group velocity, exist frequently at the high-symmetry points, *e.g.*, the saddle structure at M point, and the parabolic states at Γ center. These band-edge states will create very strong van Hove singularities,^63^ and thus, are responsible for the optical excitations.

**Fig. 3:**
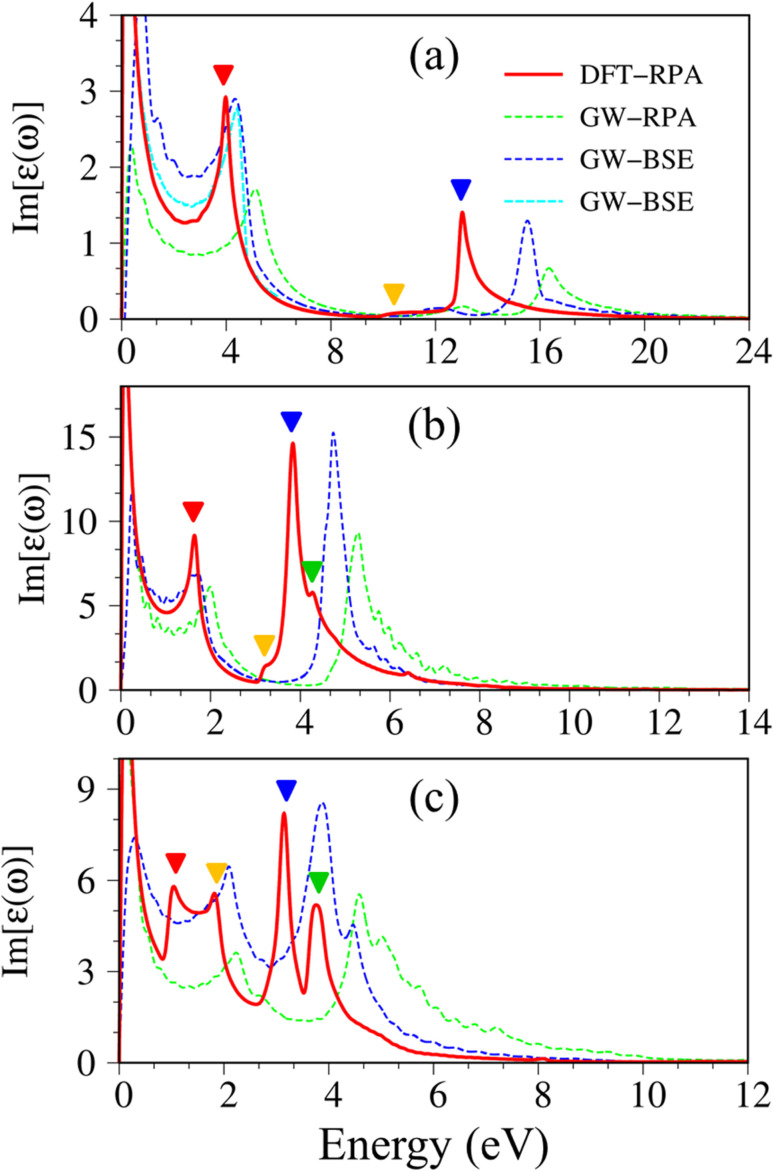
Imaginary part of dielectric functions with different levels of theory of (a), graphene, (b) silicene, and (c) germanene, respectively. Red line: DFT-RPA; dashed-green line: GW-RPA; dashed-blue line: GW-BSE; dashed-cyan line: GW-BSE calculation from ref. 24. The color triangles in figures (a)–(c) indicate the prominence of optical excitation peaks in DFT theoretical curves that correspond to the vertical arrows in Fig. 2. While the difference between the dashed-blue (GW-BSE) and the dashed-green (GW-RPA) curves indicates the excitonic effects.

There are some similarities and differences between graphene and other group IV monolayers. Both silicene and germanene display the modified Dirac cone with a smaller group velocity.^58^ The top of the valence band and the bottom of the conduction band present a slight separation. The increased SOC in the buckled honeycomb lattice^25^ is responsible for a narrow bandgap (*E*_g_ ∼ 1.55 meV and ∼23.9 meV for silicene and germanene, respectively). The band wide of the occupied energy sub-bands gradually reduces due to the weaker chemical bonding. The π and σ energy sub-bands gradually swap to each other at the wave vector space between Γ and M high symmetry points (the anti-crossing behaviors) as illustrated in the green circle in Fig. 2(d) and (h). This phenomenon reflects the sp^2^–sp^3^ hybridization in low-buckled systems. It is important to emphasize that these anti-crossing behaviors in the reciprocal space will induce strong van Hove singularities in the density of states (green arrows in Fig. 2(f) and (i)) and, thus, create prominent optical excitations (an additional peak in Fig. 3(b) and (c)).

The following notifications are used in describing the optical properties of graphene, silicene, germanene, and related systems. Because of a strong depolarization effect in these two-dimensional systems, we show below only the optical absorption for the case with the light polarization vector perpendicular to the *c*-axis (in-plane polarization). The absorbance spectra of graphene, silicene, and germanene at low frequencies (below 0.3 eV) are not discussed because intra-band transitions and temperature effects are remarkable. Because e–e interactions and e–h couplings, respectively, only cause blue and red shifts in the prominence peaks but cannot strongly modify the optical spectra in most cases, optical properties within the DFT-RPA level of theory are used to describe the connection between the initial and final states, where they can be calculated with dense *k*-grids. While the excitonic effects can be evaluated based on the similarities and differences between the GW-RPA and GW-BSE spectra.

Pristine graphene exhibits unusual optical excitation phenomena as indicated in Fig. 3(a). The imaginary part of the frequency-dependent bare response function, Im[*ε*(*ω*)], shows three prominent absorption structures, respectively, at 4.91 eV, 9.74 eV, and 12.90 eV. The featured results reflect the different orbital hybridizations of the 2D carbon honeycomb crystal (Table 2). The first, the second, and the third peaks, respectively, arising from the inter-π-band transitions associated with the saddle M-point [the red arrow in Fig. 2(a)], the excitations of the σ band from the Γ point [the yellow arrow in Fig. 2(a)], and the excitations of the σ band from the M point [the blue arrow in Fig. 2(a)]. Such identifications are delicately achieved from the orbital-decomposed van Hove singularities; that is, the initial and/or the final states must possess the singular densities of states. It is very important to note that the transitions between the π and σ states are absent even though their joint density of states is significant. This is due to the vanishing of the in-plane matrix element |〈*φ*_*xy*_|p_*xy*_|*φ*_*z*_〉|^2^ in the planar honeycomb lattice. The conclusion is in good agreement with previous publications.^65,66^

Monolayer silicene, which has weak sp^3^ bondings (the low buckling crystal), can generate an optical transition efficiency within a narrow window below 8 eV (Fig. 3(b)), being much smaller than that of graphene. This directly reflects the larger bond lengths and the relatively weak σ bondings. The magnitude of the dielectric functions becomes stronger, *e.g.*, the maxima of Im[*ε*(*ω*)], respectively, corresponding to ∼4 eV and ∼2 eV for graphene (Fig. 3(a)) and silicene (Fig. 3(b)). Such behavior is closely related to the sum rule of the total single-particle excitations (the total charge density).^67^ Silicene shows four singular structures. The first peak at 1.90 eV (red arrow) arises from the inter-π-band transitions of Si-3p_*z*_ orbitals near the M saddle, the second peak at 3.78 eV (yellow arrow) is related to the σ electron–hole excitations at the Γ center, and the third prominence structure at 3.94 eV (blue arrow) is due to the σ-electronic excitation channel through (3p_*x*_, 3p_*y*_) → (3p_*x*_, 3p_*y*_). Very interestingly, an additionally prominent peak at 4.19 eV is due to the dominating orbitals of (3p_*x*_, 3p_*y*_, 3p_*z*_) → (3p_*x*_, 3p_*y*_, 3p_*z*_). Such structures are purely induced by the band-edge states under the π and σ anti-crossing behaviors (the vertically green arrow in Fig. 2(d)), since the joint density of states corresponding to these valence and conduction energy sub-bands is sufficiently large for the hole and electron energy spectra (Fig. 2(f)). That is to say, this is obvious evidence of the non-perpendicular π and σ in a buckled Si-honeycomb crystal.

The above-mentioned optical excitation phenomena of silicene are also revealed in germanene, such as the single-particle absorption structures at 1.02 eV are due to the (4p_*x*_, 4p_*y*_) → (4p_*x*_, 4p_*y*_) excitation channels from the Γ point in Fig. 3(g), the prominence peak at 1.87 eV originated from the 4p_*z*_ → 4p_*z*_ transitions associated with the saddle M point. The prominence adsorption structure at higher frequency belongs to the (4s, 4p_*x*_, 4p_*y*_) → (4s, 4p_*x*_, 4p_*y*_) transition at the M point. An additionally prominent peak (green arrow in Fig. 3(c)) that belongs to anti-crossing band edge states (4p_*x*_, 4p_*y*_, 4p_*z*_) → (4p_*x*_, 4p_*y*_, 4p_*z*_) located at 3.68 eV. This peak is more obvious compared with that of silicene since the joint density of states related to this transition is larger under the stronger sp^3^ hybridizations.

**Fig. 4:**
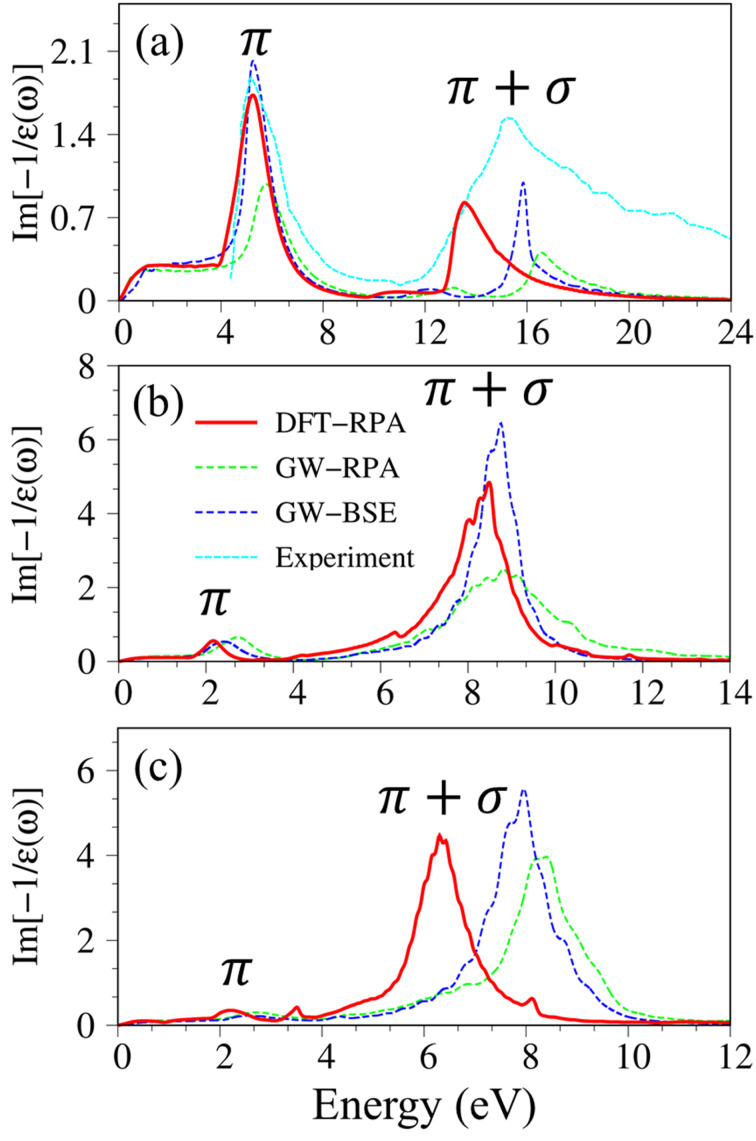
The energy loss functions with different levels of theory of (a) graphene, (b) silicene, and (c) germanene, respectively. Red line: DFT-RPA; dashed-green line: GW-RPA; dashed-blue line: GW-BSE. Experiment data of free-standing graphene^64^ are included by the dashed-cyan line in (a).

After the charge screenings of all valence electrons, the energy loss functions 
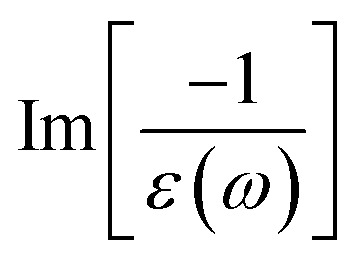
 of graphene [Fig. 4(a)] reveal the coherent features of charge oscillations.^68,69^ The π plasmon and the π + σ plasmon, respectively, survive 5.71 eV, and 14. 80 eV. Apparently, these energy-dependent plasmons belong to the quantization modes of the collective excitations of π and all valence electrons under the long wavelength limit. Such collective excitations could co-exist with the prominent Landau dampings of the excited electron–hole pairs. These two kinds of quasiparticle excitations cooperate to present the prominent absorption coefficient within the frequency range of *ω* < 20 eV. There also exists a drastic decline near the plasmon frequencies for the frequency-related absorbance and reflectance spectra [Fig. S2(a) and S3(a)†]. Very interestingly, *R*(*ω*) totally vanishes within the specific range 6 eV < *ω* < 12 eV. Most of the incident light is transmitted through single-layer graphene where only a partial perturbation is absorbed by material [transferred to heat reservoir]. Concerning the many-body effects, the results of GW + RPA point out a large blue shift of both π and π + σ coherent excitations, while the results of BSE on the top of GW functions cancel this effect. The energy and relative intensity of the π and the π + σ plasmon modes under GW + BSE approximation agree well with current experimental measurements (dashed-cyan curve).^64,70^

Silicene and germanene can be distinguished from graphene by the reduction of π and σ bands, as well as significant sp^2^–sp^3^ hybridizations. In the case of silicene, the screened response function [Fig. 4(b)], the absorbance, and the reflectance spectrum [Fig. S2(b) and S3(b)†] exhibit the plasmon peaks and edges at *ω*_P_ ∼2.37 eV and 8.83 eV. The former and the latter belong to the π-like and π + σ plasmon modes of 3p_*z*_ and [3s, 3p_*x*_, 3p_*y*_] orbitals, respectively. Compared with that of graphene, such plasmon modes undergo a significant redshift; furthermore, the relative intensity of π-like plasmon mode is generally smaller than that of the π + σ plasmon. This phenomenon is different from the intrinsic π plasmon mode in graphene and can be ascribed to the weakening of the π bonding in the non-planar layer due to the mix of the π and σ orbitals.^71^ The purely π-like plasmon modes almost disappear in the energy loss functions of germanene because of strong sp^3^ hybridizations. The intensity and frequency of plasmon modes are directly related to the absorption and reflection spectra of germanene [Fig. S1(c) and S2(c)†]. For example, the drastic decline of reflection spectra and the vanishing of absorption coefficient beyond the plasma frequency.

Atomic thin 2D materials are expected to have strong and special excitonic effects owing to two important factors: (i) quantum confinements make the excited electrons and excited holes closer, and (ii) the absence of vertical electronic screenings induces stronger Coulomb interactions. These two well-defined factors will be responsible for the formation of excitonic resonances or robust exciton bound-states with remarkable binding energies. To evaluate the excitonic effects, we calculate the optical excitations with and without excitonic effects but include self-energy effects (the dashed-blue GW-BSE, and dash-green GW-RPA, respectively in Fig. 3(a)). After including excitonic effects, the optical spectrum of graphene shows a noticeable red-shift and drastic change in the shape of the first (∼600 meV red-shift^24^), second (∼400 meV red-shift), and third (∼1 eV red-shift) optical excitations. Especially for the latter one with its effect being almost stronger than that of the formers. Generally, the excitonic effects of graphene are much stronger than that of graphite bulk counterpart^72^ and comparable with current theoretical calculations.^24^ However, graphene cannot create any bound state excitons and thus, the couple of electron–hole pairs are very weak. Similar behaviors are also found in silicene and germanene. This unusual quasiparticle phenomenon could be attributed to the zero-gap/narrow-gap semiconducting properties.

Graphene, silicene, and germanene have a distinct and wide absorbance spectrum, which suggests that they might be used for optoelectronic applications and photo-related applications.

### 3.2 Graphene, silicone, and germanone

**Fig. 5:**
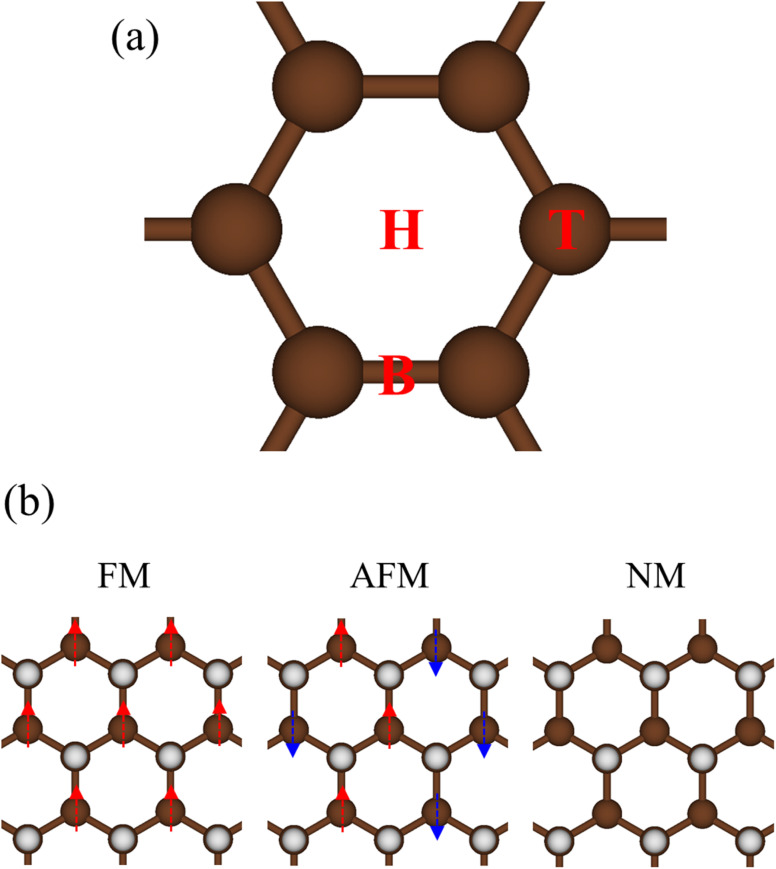
(a) The possible adsorption sites are labeled H, T, and B for the hydrogen atom at the center of the hexagon, the top of the C/Si/Ge atom, and at the bridge side in the middle of bonding, respectively. (b) Various magnetic configurations of semi-hydrogenated group IV monolayers: ferromagnetic (FM), antiferromagnetic (AFM), and nonmagnetic (NM) configurations.

**Fig. 6:**
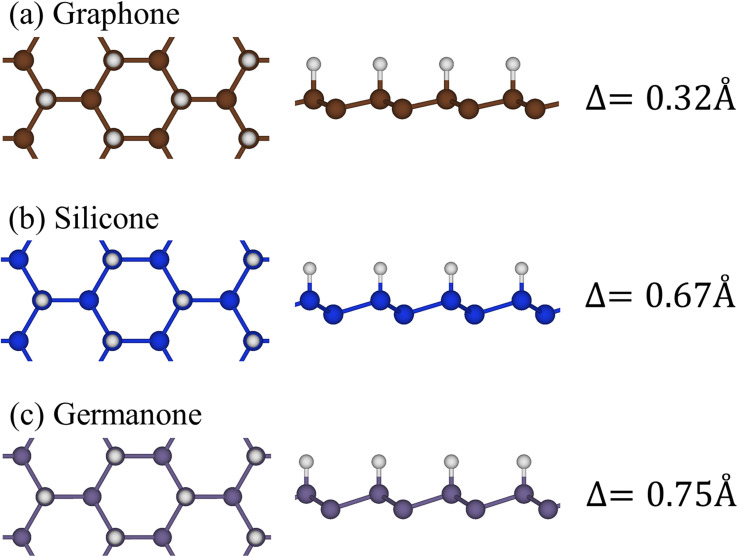
The top and side views of the geometric structure of (a) graphone, (b) silicone, and (c) germanone, respectively.

To check the most favorite side for hydrogen adsorption, we have constructed the model with several possible adsorption sides as shown in Fig. 5(a), including the top (T) of the C/Si/Ge atom, the hollow (H) of the hexagon structure, and the bridge (B) side at the middle of the chemical bonding. Nevertheless, when the hydrogen atom adsorption is on only one side of the two-dimensional sheet, the electron densities at the top and the bottom are unpair and thus can create a distinct magnetic configuration. To evaluate the referred spin alignment, we also considered three magnetic configurations as shown in Fig. 5(b): the ferromagnetic coupling (FM), the anti-ferromagnetic coupling (AFM), and the non-magnetic states (NM). The calculated results indicate that the most favorite position for H atoms adsorption is at the top site of carbon/silicon/germanium atoms, in which, the ferromagnetic phase is in the most preferred configurations. Such conclusions are good in agreement with previous calculations^73,74^(Fig. 6).

Hydrogen atom strongly hybridizes with the C, Si, and Ge^26,27^ and significantly alter the geometric, electronic, and optical properties of the honeycomb lattices. As for graphene, 1.5 Å of C–C bond lengths denoted that the planar structure is almost determined by σ bond due to the (2s, 2p_*x*_, 2p_*y*_) orbitals. However, the height difference between two sub-lattices is enhanced to be 0.34 Å, indicating the sp^2^ → sp^3^s transition. In addition, the H–C bond lengths are approximately 1.12 Å because of the strong and stable covalent σ bonds. The abovementioned bond configurations are closely related to the strong covalent bonds between 2p_*z*_ and 1s orbitals, as well as the weak sp^3^ hybridization of four orbitals (2s, 2p_*x*_, 2p_*y*_, and 2p_*z*_). Similar behaviors also occurred for hydrogenated silicene and germanene.

**Fig. 7:**
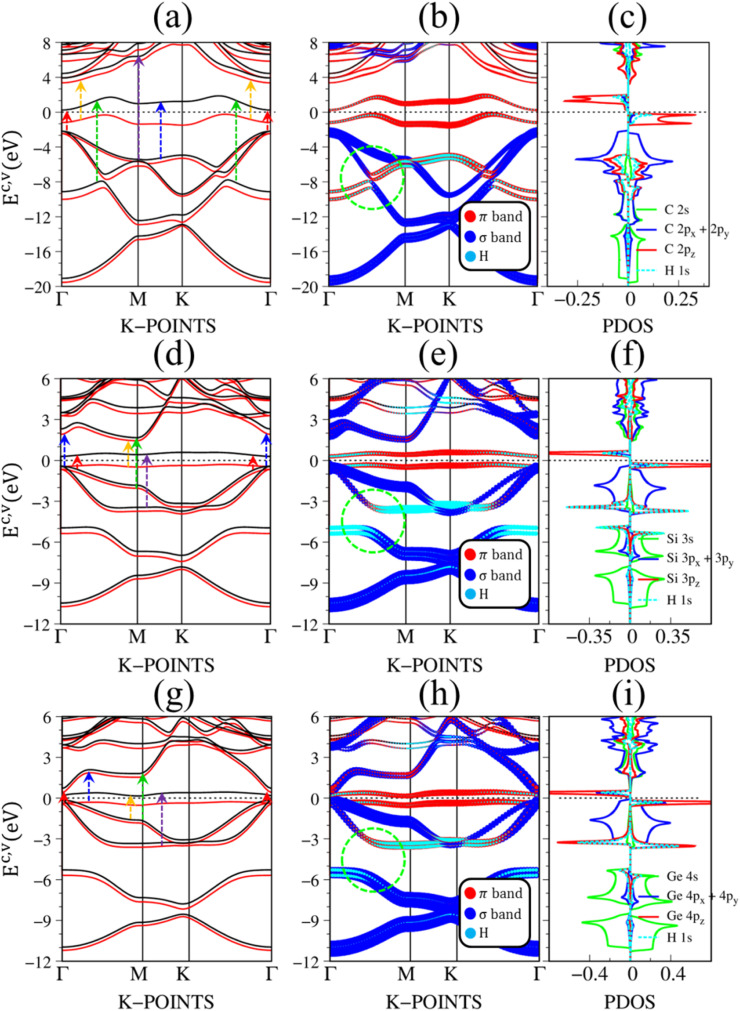
The DFT electronic band structure, the projected electronic wave functions, and the orbital-projected density of states of (a), (b), (c) graphone, (d), (e), (f) silicone and (g), (h), (i) germanone, respectively. The red and black curves in (a), (d), and (g) represent the spin-up and spin-don energy sub-bands, while the colored arrows indicate the optical excitations that correspond to the prominence absorption peaks in Fig. 8. The green circles in (b), (e), and (h) indicate the anti-crossing behaviors in graphone, silicone, and germanone.

The band structure is dramatically changed by the strong C-/Si/Ge-H chemical bondings. In the case of graphene, the absence of hexagonal symmetry destroys the Dirac-cone structure (Fig. 7(a)). The 1s orbital of each H atom strongly hybridizes with carbon 2p_*z*_ orbitals, so that the (C, H)-co-dominated bands are revealed (Fig. 7(b)). The π and σ energy sub-bands gradually swap to each other at the wave vector space between Γ and M high symmetry points as illustrated in the green circle in Fig. 7(b), this anti-crossing phenomenon directly reflects the sp^2^ → sp^3^s transition. The single-side hydrogenation belongs to an indirect semiconductor with a gap of about 0.5 eV. This value could be improved after the GW corrections are applied (see Table 1 and Fig. S4†). Very interestingly, graphone exhibits spin-dependent energy bands, as clearly shown in Fig. 7(a). The spin splitting appears in the range of |*E*^*c*,*v*^ ≤ 2 eV|, and it is almost negligible at larger state energies. For the spin-up and spin-down states, the energy bands nearest to *E*_F_ are entirely occupied and unoccupied, respectively, indicating the preferred magnetism. Silicone and germanone exhibit similar phenomena (Fig. 7(d)–(f) and (g)–(i)); however, spin-splitting around the low-energy band is narrower, indicating a smaller magnetic moment (Table 1).

**Table 1:** The geometric parameter, electronic and magnetic property, and exciton binding energy of monolayer pristine graphene, silicene, germanene, and their semi-hydrogenated systems: graphone, silicone, and germanone. The previous theoretical and experimental results are also listed

Material	X–X bond (Å)	X–H bond (Å)	Bulked (Å)	Band gap (eV)	Magnetism (*μ*_B_)	Exciton binding *E*_xb_ (eV)
Graphene	1.42	—	0	0	0	0
	1.41^a^	—	0^a^	0^a^	0^a^	
Silicene	2.24	—	0.46	0.015	0	0
	2.25^b^	—	—	—	—	
Germanene	2.43	—	0.67	0.023	0	0
	2.44^c^	—	0.73^c^	0^c^	0	
Graphone	1.49	1.16	0.32	0.44	0.33	0.62
				2.98^d^		
				2.79^e^		
				3.01^f^		
	1.50^g^	1.16^g^	0.34^g^	0.50^g^	—	
Silicone	2.34	1.51	0.67	0.59	0.32	0.51
				1.77^d^		
	2.31^h^	1.51^h^	—	—	—	
Germanone	2.48	1.57	0.75	0.32	0.45	0.26
				1.06^d^		
	2.49^i^	1.58^i^	0.76^i^	0.4^i^	—	

a Ref. 28.

b Ref. 27.

c Ref. 58.

d This work using GW-corrections.

e Ref. 59.

f Ref. 60.

g Ref. 28.

h Ref. 27.

i Ref. 61.

**Table 2:** The specific relations between the prominent absorption structures and the dominated orbital hybridizations for graphene, silicene, and germanene

Material	Energy (eV)	Colored arrow	Orbital hybridizations
Graphene	4.91	Red	2p_*z*_ → 2p_*z*_
	9.94	Yellow	(2p_*x*_, 2p_*y*_) → (2p_*x*_, 2p_*y*_)
	12.90	Blue	(2s, 2p_*x*_, 2p_*y*_) → (2s, 2p_*x*_, 2p_*y*_)
Silicene	1.90	Red	3p_*z*_ → 3p_*z*_
	3.78	Yellow	(3p_*x*_, 3p_*y*_) → (3p_*x*_, 3p_*y*_)
	3.94	Blue	(3s, 3p_*x*_, 3p_*y*_) → (3s, 3p_*x*_, 3p_*y*_)
	4.19	Green	(3p_*x*_, 3p_*y*_, 3p_*z*_) → (3p_*x*_, 3p_*y*_, 3p_*z*_)
Germanene	1.02	Yellow	(4p_*x*_, 4p_*y*_) → (4p_*x*_, 4p_*y*_)
	1.87	Red	4p_*z*_ → 4p_*z*_
	3.24	Blue	(4s, 4p_*x*_, 4p_*y*_) → (4s, 4p_*x*_, 4p_*y*_)
	3.68	Green	(4p_*x*_, 4p_*y*_, 4p_*z*_) → (4p_*x*_, 4p_*y*_, 4p_*z*_)

Hydrogenated systems, with an unusual electronic structure,^26,27^ clearly reveal the rich optical transitions in the bare dielectric functions,^75^ screened energy loss spectra, absorption coefficients, and reflectance spectra.^76^ Compared with those of a pristine case, there exist more prominent absorption structures, directly reflecting the featured band structure after hydrogen ad-atom chemisorption. The concise orbital hybridizations and spin-polarizations of the initial valence and final conduction states, being responsible for all significant transitions, are thoroughly examined from the consistent physical quantities, in which the atom-dominated energy spectrum, the spin-dependent projected density of states, and the strong absorption peaks are unified together under a quasiparticle framework. The evaluated results are clearly shown in Table 3, being assisted by the various vertical arrows of the featured band structure and the distinct excitation channels cover the pronounced absorption peaks. It is very important to notice that the different spin transitions are forbidden due to the lack of required spin flip under photon absorption.^77^

**Table 3:** The specific relations between the prominent absorption structures and the dominated orbital hybridizations & spin-polarizations for graphone, silicone, and germanone

Material	Energy (eV)	Colored arrow	Orbital hybridizations	Spin
Graphone	2.52	Red	1s + (2p_*x*_, 2p_*y*_) → 1s + (2p_*x*_, 2p_*y*_)	↓
	4.45	Yellow	1s + (2p_*x*_, 2p_*y*_, 2p_*z*_) → (2p_*x*_, 2p_*y*_, 2p_*z*_)	↑
	6.45	Blue	1s + 2p_*z*_ → 2p_*z*_	↓
	9.36	Green	1s +2p_*z*_ → 1s + 2p_*z*_	↓
	11.89	Purple	1s + (2p_*x*_, 2p_*y*_, 2p_*z*_) → (2p_*x*_, 2p_*y*_, 2p_*z*_)	↑ + ↓
Silicone	0.89	Red	(3p_*x*_, 3p_*y*_) → 1s + (3p_*x*_, 3p_*y*_)	↓
	2.35	Yellow	1s + (3p_*x*_, 3p_*y*_, 3p_*z*_) → (3p_*x*_, 3p_*y*_, 3p_*z*_)	↑
	3.13	Blue	(3p_*x*_, 3p_*y*_) → (3p_*x*_, 3p_*y*_)	↑ + ↓
	3.45	Green	1s + (3p_*x*_, 3p_*y*_, 3p_*z*_) → (3p_*x*_, 3p_*y*_, 3p_*z*_)	↑ + ↓
	4.01	Purple	1s + (3p_*x*_, 3p_*y*_, 3p_*z*_) → (3p_*x*_, 3p_*y*_, 3p_*z*_)	↓
Germanone	0.35	Red	(4p_*x*_, 4p_*y*_) → 1s + (4p_*x*_, 4p_*y*_)	↓
	1.03	Yellow	1s + (4p_*x*_, 4p_*y*_, 4p_*z*_) → (4p_*x*_, 4p_*y*_, 4p_*z*_)	↑
	1.82	Blue	(4p_*x*_, 4p_*y*_) → (4p_*x*_, 4p_*y*_)	↑ + ↓
	3.62	Green	(4p_*y*_, 4p_*z*_) → (4p_*y*_, 4p_*z*_)	↑ + ↓
	3.98	Purple	1s + 4p_*z*_ → 1s + 4p_*z*_	↓

**Fig. 8:**
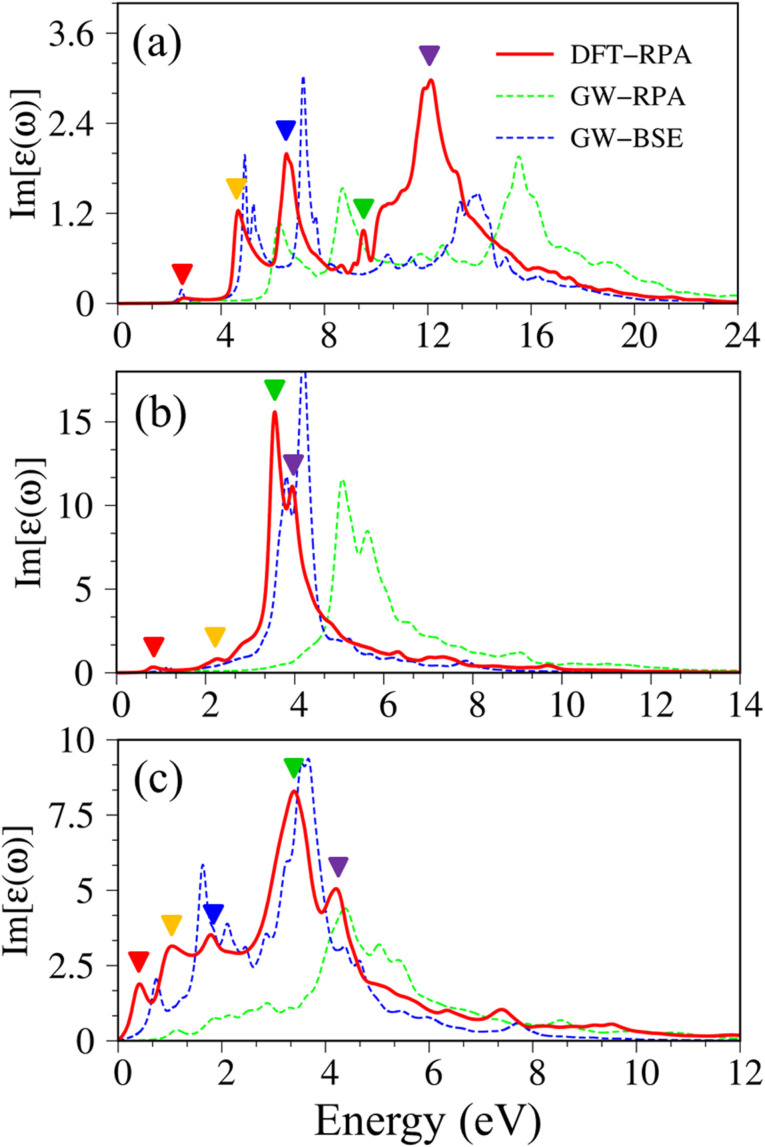
Imaginary part of dielectric functions with different levels of theory of (a) graphone, (b) silicone, and (c) germanone, respectively. Red line: DFT-RPA; dashed-green line: GW-RPA; dashed-blue line: GW-BSE. The color triangles in (a)–(c) indicate the prominence peaks in DFT optical spectra that correspond to the vertical optical excitations in Fig. 7. While the difference between the dashed-blue (GW-BSE) and the dashed-green (GW-RPA) curves indicates the excitonic effects.

For graphone, the optical gap is situated at 2.52 eV and significantly larger than the electronic band gap (0.5 eV). This is due to the opposite spin orientation and thus the promotion of electrons from the highest occupied to the lowest unoccupied states is forbidden. This rapid oscillation originates from the direct transition at the Γ point of the spin-down states between 1s + (2p_*x*_, 2p_*y*_) → 1s + (2p_*x*_, 2p_*y*_) orbitals (the red arrow in Fig. 8(a)). Beyond this threshold frequency, the absorption spectra also show four extra prominent peaks. The strong excitation at 4.45 eV (denoted by the yellow arrow) is caused by the vertical transitions of the first spin-up valence and the second spin-up conduction sub-bands of 1s + 2p_*z*_ → 2p_*z*_ orbitals. The other observable optical excitation at 6.45 eV (denoted by the blue arrow) belongs to 1s + 2p_*z*_ → 2p_*z*_ transitions of spin-down states. The weak but important transition at 9.36 eV (assigned by the green arrow) originated from the transition between 1s + 2p_*z*_ orbitals of spin-up states. The strongest optical excitation with the highest frequency at 11.92 eV (denoted by the purple arrow) promotes from 1s + (2p_*x*_, 2p_*y*_, 2p_*z*_) → (2p_*x*_, 2p_*y*_, 2p_*z*_) of both spin-up and spin-down states.

**Fig. 9:**
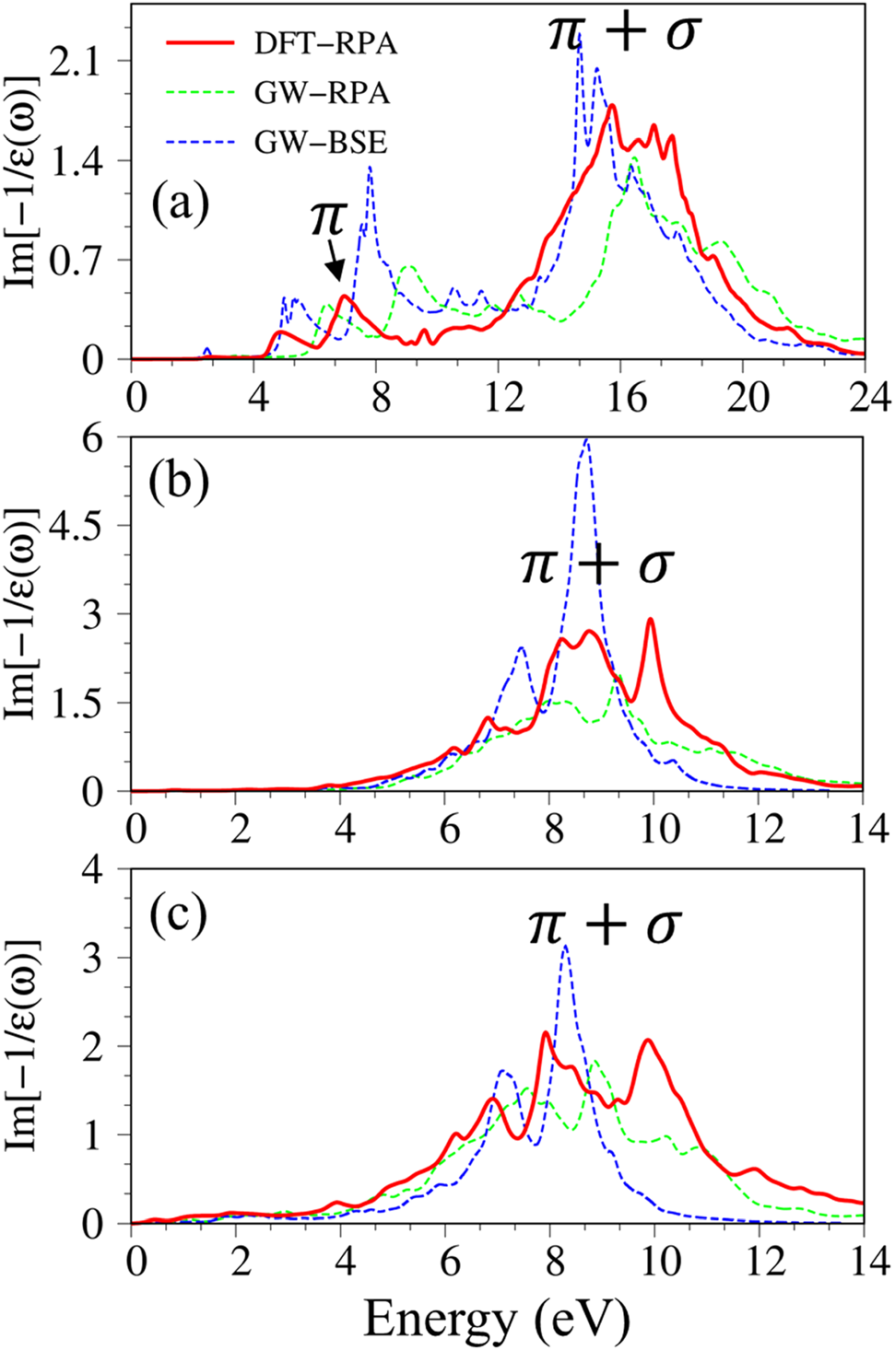
The energy loss functions with different levels of theory of (a) graphene, (b) silicene, and (c) germanene, respectively. Red line: DFT-RPA; dashed-green line: GW-RPA; dashed-blue line: GW-BSE.

Considering the collective charge screening, the energy loss functions 
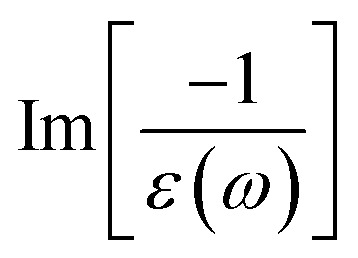
, exhibit a lot of prominent peaks (Fig. 9(a)). These structures originated from vertical excitations with different degrees of Landau damping. Roughly speaking, there exist two well-characterized collective excitations; that is, the π-like plasmon mode and σ plasmon mode at 7.6 eV and 15.7 eV, correspondingly. In which, the former is gradually suppressed owing to the transformation of sp^2^ → sp^3^s hybridizations. The purely π-like plasmons almost disappear in both silicone (Fig. 9(b)) and germanone (Fig. 9(c)) due to the partly termination of the π orbitals and enhanced buckling in hydrogenated systems.

**Fig. 10:**
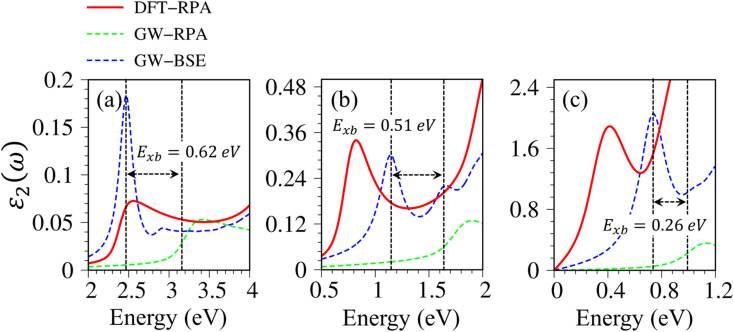
The enlargement of the imaginary parts of dielectric functions with different levels of theory of (a) graphone, (b) silicone, and (c) germanone, respectively. Red line: DFT-RPA; dashed-green line: GW-RPA; dashed-blue line: GW-BSE.

Very interestingly, hydrogenated systems exhibit significant excitonic effects in terms of the number, frequency, intensity, and form of prominent absorption structures. Concerning graphone (Fig. 10(a)), an additional peak located at 2.5 eV manifests the excitonic features because the optical absorption without electron–hole couplings nearly vanishes in the interval of 0 to 3 eV. The properties of this sharp peak could be understood as the vertical promotion between the last spin-down occupied states and the first spin-down unoccupied states at the Γ point. The exciton binding energy (*E*_xb_), which is determined by the energy difference of the optical gap in cases with and without excitonic effects, is about 0.6 eV. This value is comparable with other excitonic materials, such as transition metal dichalcogenides.^78,79^ The stability of this exciton state could be attributed to the quantum confinement, low charge screening abilities [sizable electronic band gap], and the effective mass of excited electrons and excited holes [the inverse of band curvatures]. Compared with silicone and germanone, the exciton binding energy lies in the following other: graphone > silicone > germanone. Of course, the composite quasiparticles in hydrogenated systems are quite stable, so they are expected to survive at room temperature.

**Fig. 11:**
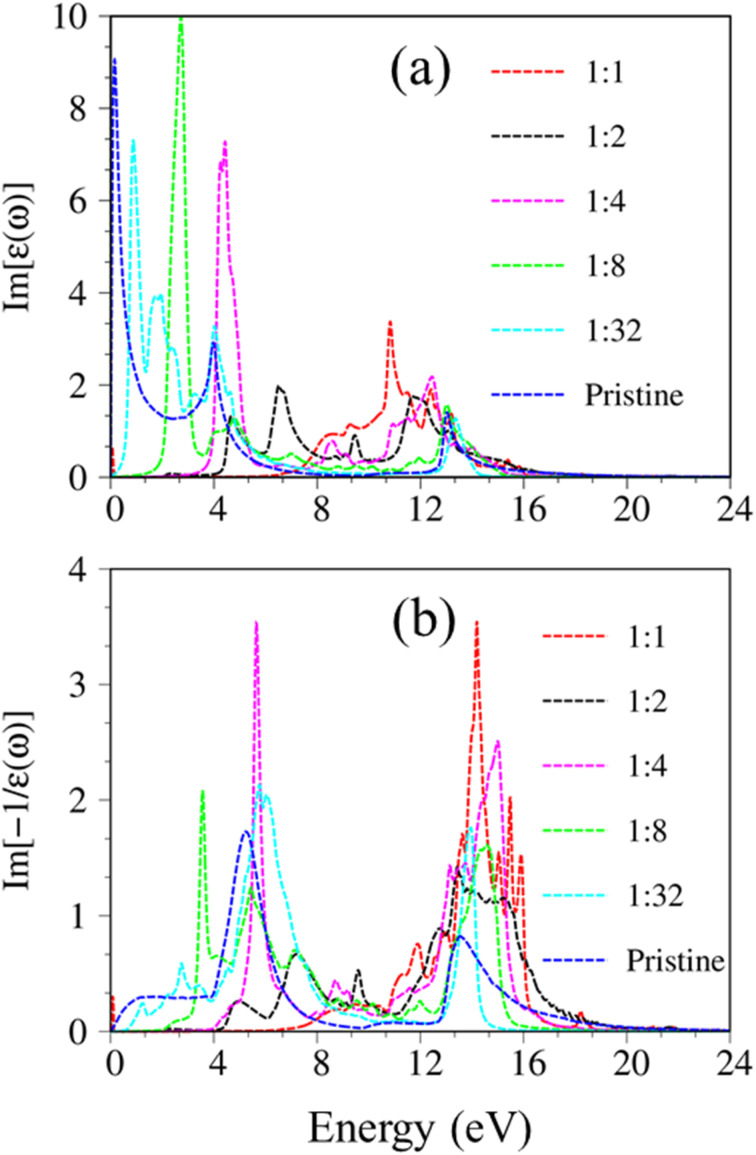
(a) The imaginary part of dielectric functions and (b) energy loss functions of hydrogenated graphene under various hydrogen concentrations (all calculations are based on the DFT level of theory).

The last remark concerns the optical characteristics of group IV monolayers as hydrogen concentration variations. Using graphone as an example, as hydrogen concentration decreases, sp^3^s hybridization in graphone progressively recovers to sp^2^ one in pure graphene (Fig. S9†). The low-lying energy band is built from reform π and π* bonding, the energy gap decreases and fluctuates with the hydrogen concentration as illustrated in Fig. S10 and S11.† As a result, the main properties of single-particle excitations and collective excitations, such as optical gap and features of π and σ plasmon modes, are strongly dependent on hydrogen concentration and adsorption position (Fig. 11). Very interestingly, the optical features of graphone recovered back into the pristine condition for a very dilute hydrogen concentration case, *e.g.*, H : C = 1 : 32. With decreasing hydrogen concentration, the semiconductor nature of graphone gradually transfer back into the semi-metallic one, and hence the excitonic effects are expected to be greatly diminished. The adsorption of hydrogen atoms on group IV monolayer surfaces demonstrates efficient ways to control the electrical, magnetic, and optical properties of such materials. The current theoretical studies have provided a perspective on the relation of orbital hybridizations and spin-orbital polarization and the electronic, optical properties of monolayer group-IV-related systems. The intriguing properties of these materials may be useful for a variety of high-tech applications, including electrical, optoelectronic, spintronic, and hydrogen storage applications.

The theoretical predictions in the current research could provide useful information for the experimental measurements. Up to now, the information about the optimal geometric, electronic, and optical properties of graphene-like related systems could be confirmed by high-technique equipment such as X-ray photoelectron spectroscopy (XPS),^80^ scanning tunneling microscopy (STM),^81^ scanning tunning spectroscopy (STS),^82^ scanning tunning electron microscopy (STEM),^83^ angle-resolved photoemission spectroscopy (ARPES),^84^ photoluminescence spectroscopy (PL)^85^ and other optical measurements.^86^ Typically, XPS is a powerful technique to determine how many atoms are attached to the group IV monolayer and types of dopants, *e.g.*, the estimated concentration for oxidized carbons and graphitic carbons.^87^ The top-view information and the atomic arrangement of monolayer group IV materials and their semi-hydrogenated systems could be detected by the STS and STEM. The occupied electronic properties such as energy dispersion and energy bandwidth can be carried out by using the ARPES measurement, while the STS technique could be used to measure the electronic band gap and the van Hove singularities in the vicinity of the Fermi energy. Such measurements have been established for both graphene^88–90^ and it's hydrogenated systems.^91,92^ In addition, the exciton energy and the reflectance, absorbance spectra could be detected *via* PL, reflectance, and absorbance measurements, respectively. Overall, the theoretical results in our work are in good agreement with previous theoretical predictions and experimental measurements.

## 4 Conclusions and remarks

Based on the first-principles calculations, the geometric, electronic, and optical properties of group-IV-related systems are investigated. Graphene, silicene, germanene, and their semi-hydrogenated systems exhibit unique and diverse physical properties. In particular, the orbital hybridizations between X–X and X–H atoms, and the spin interactions are the critical factors affecting the optimized geometric structures, the electronic properties, the optical excitations, and excitonic effects. Our results are consistent with earlier theoretical calculations and experimental measurements.

Graphene, and silicene/germanene, respectively, belong to the sp^2^, and sp^2^–sp^3^ hybridizations. Surviving weak sp^3^ hybridization has considerable impacts on the electronic characteristics and optical excitations in two-dimensional materials. For example, silicene and germanene present the anti-crossing phenomena in the electronic band structure, the corresponding van Hove singularities in the density of states, the extra prominent optical excitations in the imaginary part of dielectric functions, and the suppression of π-like plasmon mode in the energy loss functions. Such interesting phenomena originated from the weakening of the σ bondings and partial hybridizations of π and σ orbitals in the low-buckled structures.

Due to the strong couplings of p_*z*_ and H-1s orbitals, a lot of significant characteristics of graphone, silicone, and germanone are revealed in the first-principles calculations, including a sizable electronic band gap, a destroyed linear and isotropic Dirac cone, the non-crossing-and-anti-crossing-phenomena and the critical points/band edge states (van Hove singularities), the presence of spin-split, spin-degeneracy energy band structure and the significant magnetic moments. The frequency-dependent optical properties, the transverse dielectric functions, energy loss functions, reflectance spectra, and absorption coefficients present unusual spin-dependent optical excitations and distinct plasmon modes. The stable exciton states are also predicted to survive in semi-hydrogenated group IV monolayers. The current study is of paramount importance not only for fundamental physics but also for technical applications, *e.g.*, electronic, optoelectronic, spintronic, and hydrogen storage applications.

## Conflicts of interest

The authors declare no competing interests.

## Supplementary Material

RA-012-D2RA06722F-s001
