# Supplementary material for: Electronic and optical properties of graphene, silicene, germanene, and their semi-hydrogenated systems
Source: RSC Adv. 2022 Dec 6;12(54):34851–65. doi: 10.1039/d2ra06722f (PMC9724213; doi:10.1039/d2ra06722f)
Supplement: RA-012-D2RA06722F-s001 [file RA-012-D2RA06722F-s001.pdf]

Electronic Supplementary Material (ESI) for RSC Advances.
This journal is © The Royal Society of Chemistry 2022

SUPPORTING INFORMATION

Electronic and optical properties of graphene, silicene, germanene, and their semi-hydrogenated systems

Vo Khuong Dien,[1,] Wei Bang-Li[1], Kuang-I Lin[2], Nguyen Thi Han,[1*], and Ming-Fa Lin [1,3,*]

[1]*Department of Physics, National Cheng Kung University, 701 Tainan, Taiwan*

[2]*Core Facility Center, National Cheng Kung University, 701 Tainan, Taiwan*

[3]*Hierarchical Green-Energy Material (Hi-GEM) Research Center, National Cheng Kung University, 701 Tainan, Taiwan*

*Email: han.nguyen.dhsptn@gmail.com and mflin@mail.ncku.edu.tw

1. Computational details

Based on the ground-state Kohn–Sham wave functions and the corresponding eigenvalues (E_{KS}) of the DFT level [1], the quasiparticle energy spectrum was obtained by using the GW approximation (G_0W_0) [2] on the exchange–correlation self-energy. The screening effects are described by using the plasmon-mode model of Hybertsen and Louie [3]. These approaches adopt a cutoff energy of 500 eV for expansion of plane waves and a cutoff energy of 250 eV for the response functions. The Brillouin zone was integrated with a special k-point mesh of 50 × 50 × 1 in the Γ sampling technique.

The presence of exciton states may have a significant impact on the optical responses [4]. The wave functions related to these bound states of electrons and holes could be expressed by using the following expression:

$$|S\rangle = \sum_{ks}\sum_{v}^{hole}\sum_{c}^{elec} A_{vck}^{s}|vck\rangle;$$

in which, the amplitude A_{vck}^{s} is determined by solving the standard Bethe-Salpeter equation (BSE) [5]

$$\left(E^{QP}_{ck} - E^{QP}_{vk}\right)A^{s}_{vck} + \sum_{v'c'k'} \langle vck \,|\, K^{eh} \,|\, v'c'k' \rangle A^{s}_{vck} = \Omega^{s} A^{S}_{vck},$$

where E^{QP}_{ck} and E^{QP}_{vk}, respectively, are the quasi-particle energies of the valence and the conduction states as obtained with the GW method. K^{eh} is the kernel describing the correlated electron-hole pairs, and $\Omega^{s}$is the energy of the excited states. The imaginary part of the dielectric function $\epsilon_2(\omega)$ is calculated from the excitonic states as:

$$\epsilon_2(\omega) \propto \sum_{S} \left| \sum_{cvk} A^{s}_{vck} \langle vk \,|\, v \,|\, ck \rangle \right|^2 \delta(\omega - \Omega^{s}).$$

In this part, the Tamm-Dancoff approximation (TDA) [6] was used, moreover, energy cutoff and k-point sampling are set resemblances as in the GW calculations. Lorentzian with at most 80 meV broadening was used to replace the delta function. In this work, three lowest conduction bands (CBs) and the four highest valence bands (VBs) in the Bethe-Salpeter kernel are sufficient to describe the excitonic effects.

Table S1: The adsorption energy of hydrogen on graphene, silicene, and germanene.

Materials	H side (eV)	T side (eV)	B side (eV)
Graphene	-0.433	0.403	0.229
Silicene	-3.044	-0.556	-1.812
Germanene	-2.981	-0.825	-1.712

Table S2: The total ground state energy of graphone, silicone, and germanone with various magnetic configuration.

Materials	FM (eV)	AFM (eV)	NM (eV)
Graphone	-19.871	-19.832	-19.647
Silicone	-13.552	-13.536	-13.389
Germanone	-11.964	-11.949	-11.848

Table S3: Effective mass for holes at the VBM and electrons at the CBM of graphone, silicone, and germanone.

Materials	Holes at VBM		Electrons at CBM	
	$\Gamma \rightarrow M$	$\Gamma \rightarrow K$	$\Gamma \rightarrow M$	$\Gamma \rightarrow K$
Graphone	2.14	3.47	7.18	2.67
Silicone	15.87	15.85	15.78	15.73
Germanone	17.74	17.65	17.52	17.4

2. Graphene, silicene and germanene

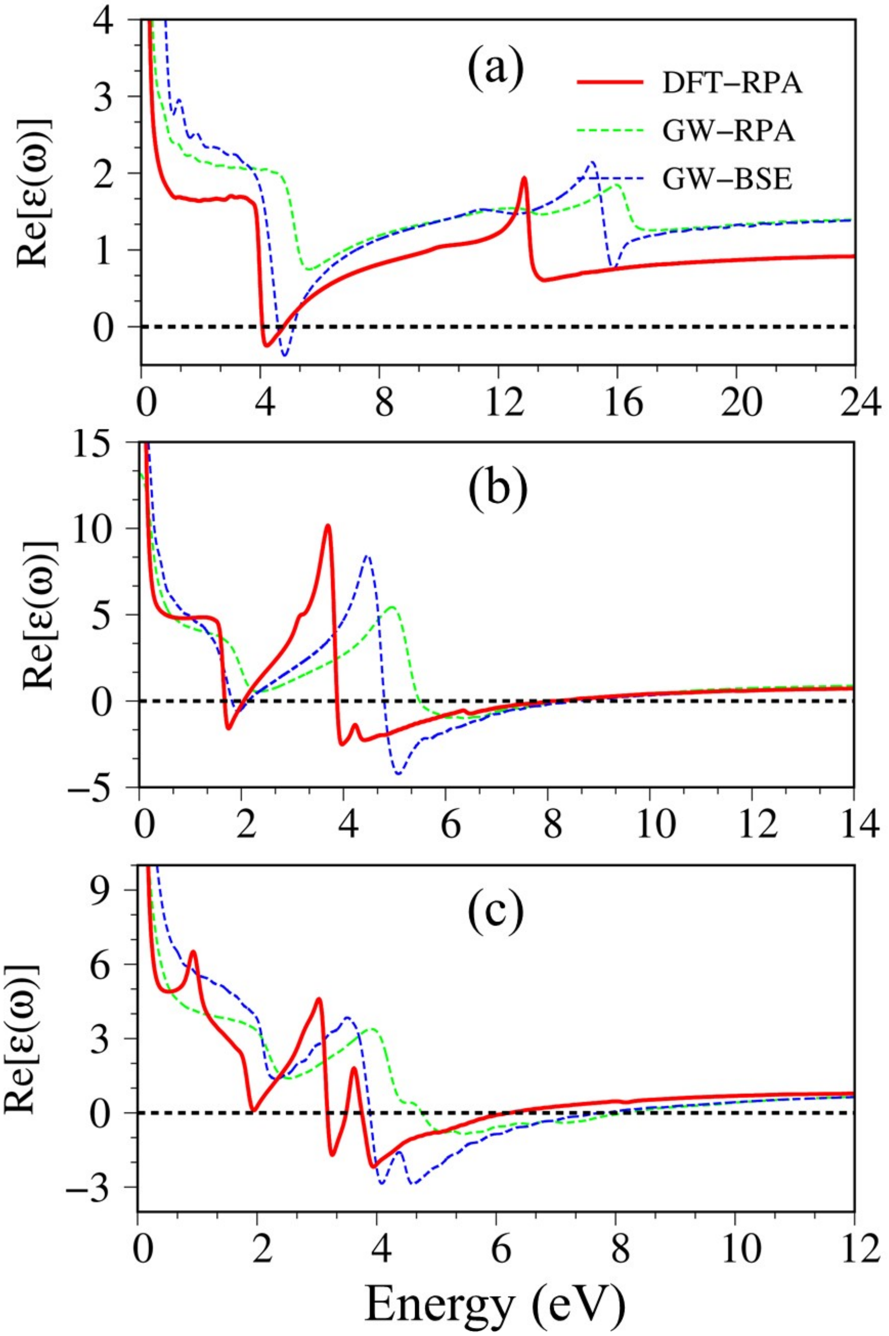

Figure S1: Real part of dielectric functions with different levels of theory of (a) graphene, (b) silicene, and (c) germanene. Red line: DFT-RPA; dashed-green line: GW-RPA; dashed-blue line: GW-BSE.

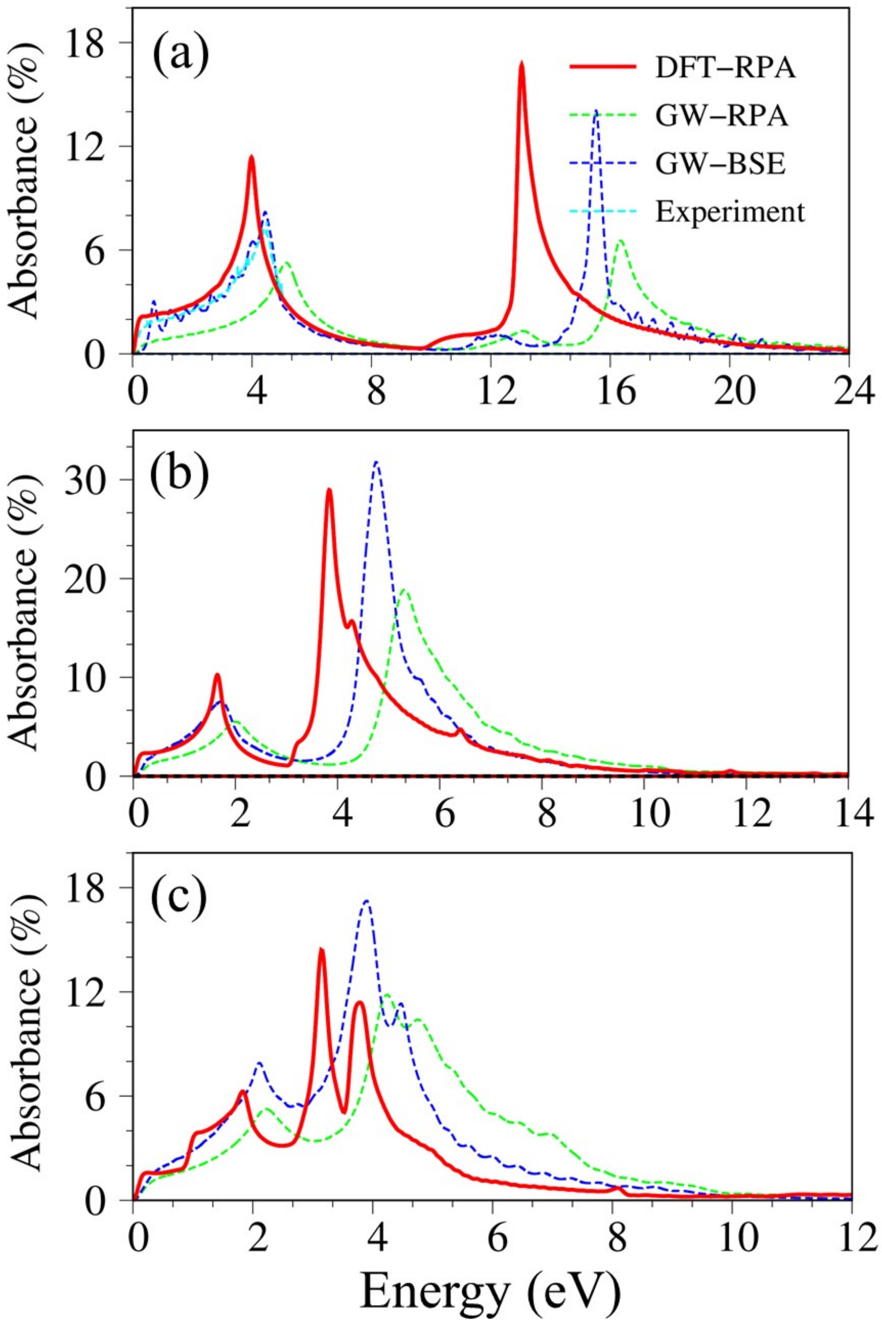

Figure S2: Absorbance coefficient with different levels of theory of (a) graphene, (b) silicene, and (c) germanene. Red line: DFT-RPA; dashed-green line: GW-RPA; dashed-blue line: GW-BSE. Experiment data of free-standing graphene [7] are included by the dashed-cyan line in (a).

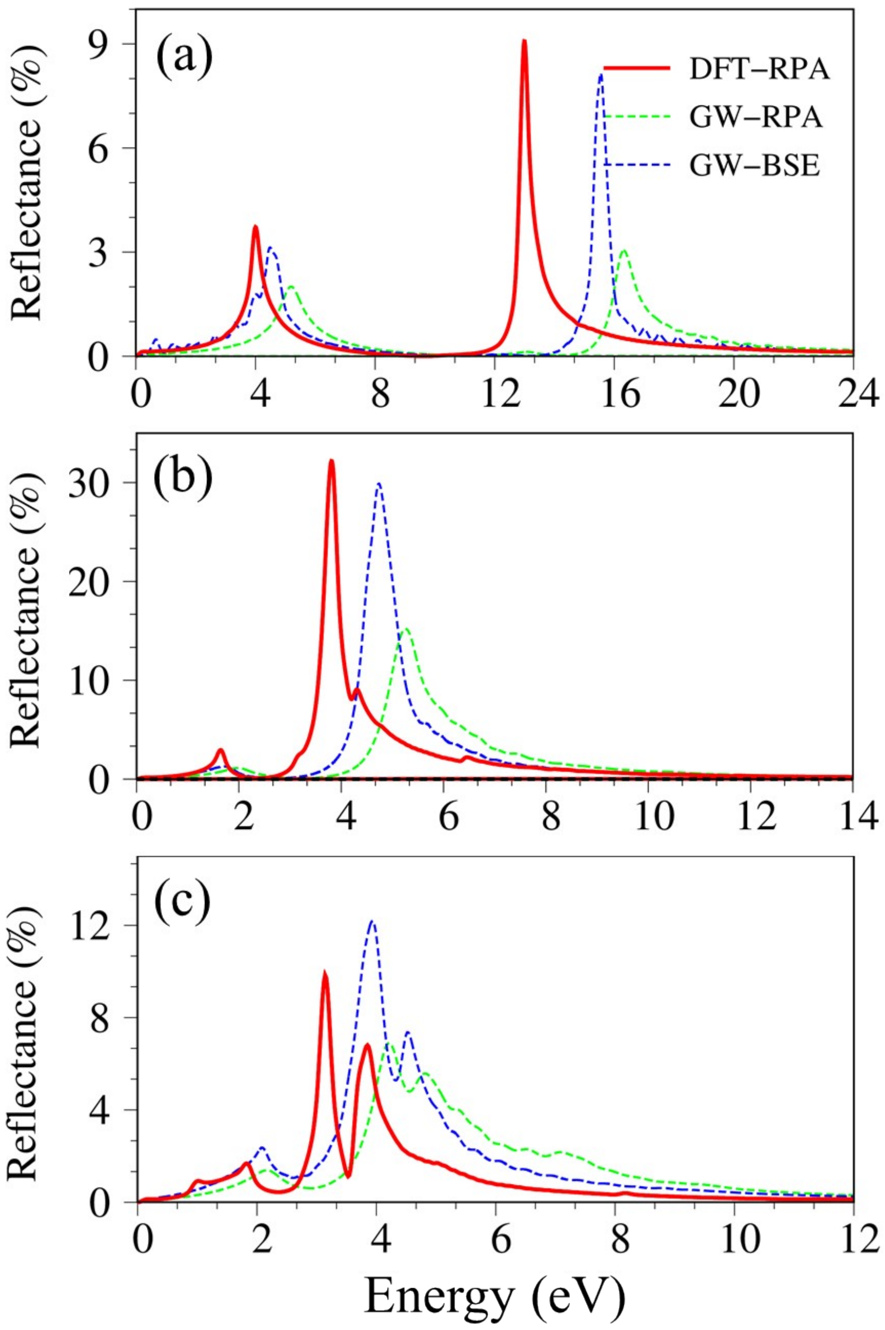

Figure S3: Reflectance coefficient with different levels of theory of (a) graphene, (b) silicene, and (c) germanene. Red line: DFT-RPA; dashed-green line: GW-RPA; dashed-blue line: GW-BSE.

3. **Germanone, silicone, and germanone**

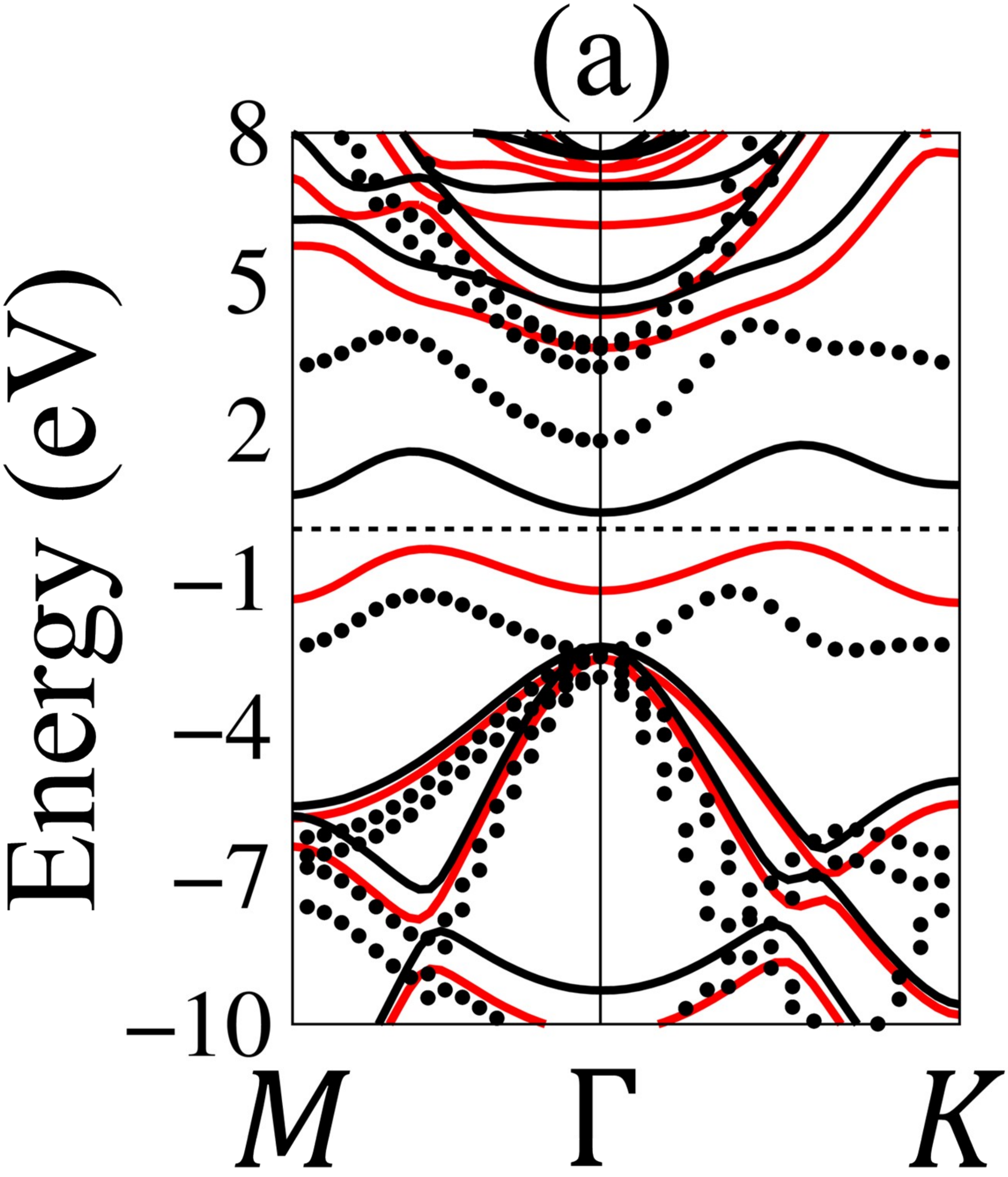

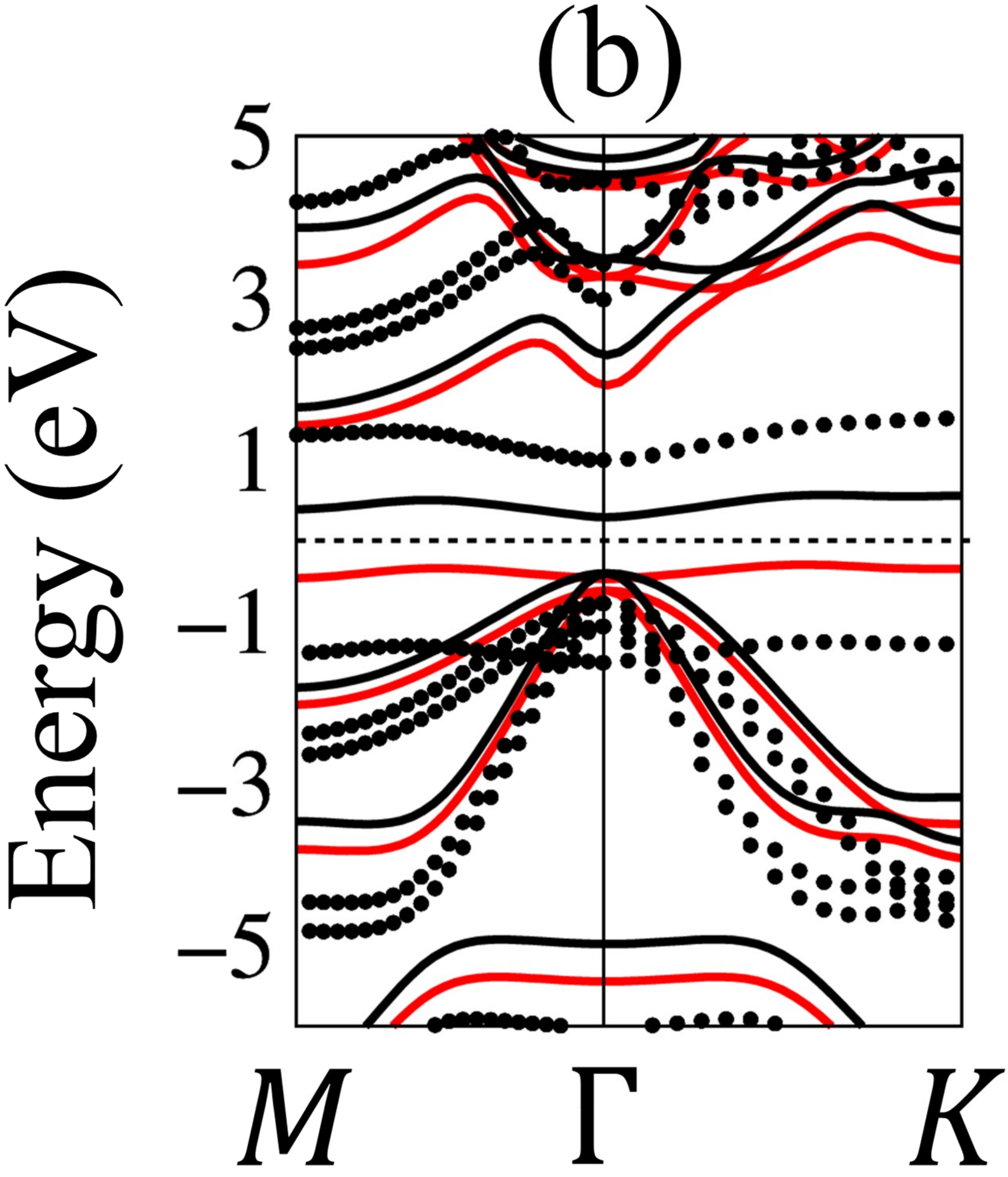

(b)
Energy (eV)
5
3
1
−1
−3
−5
M
Γ
K

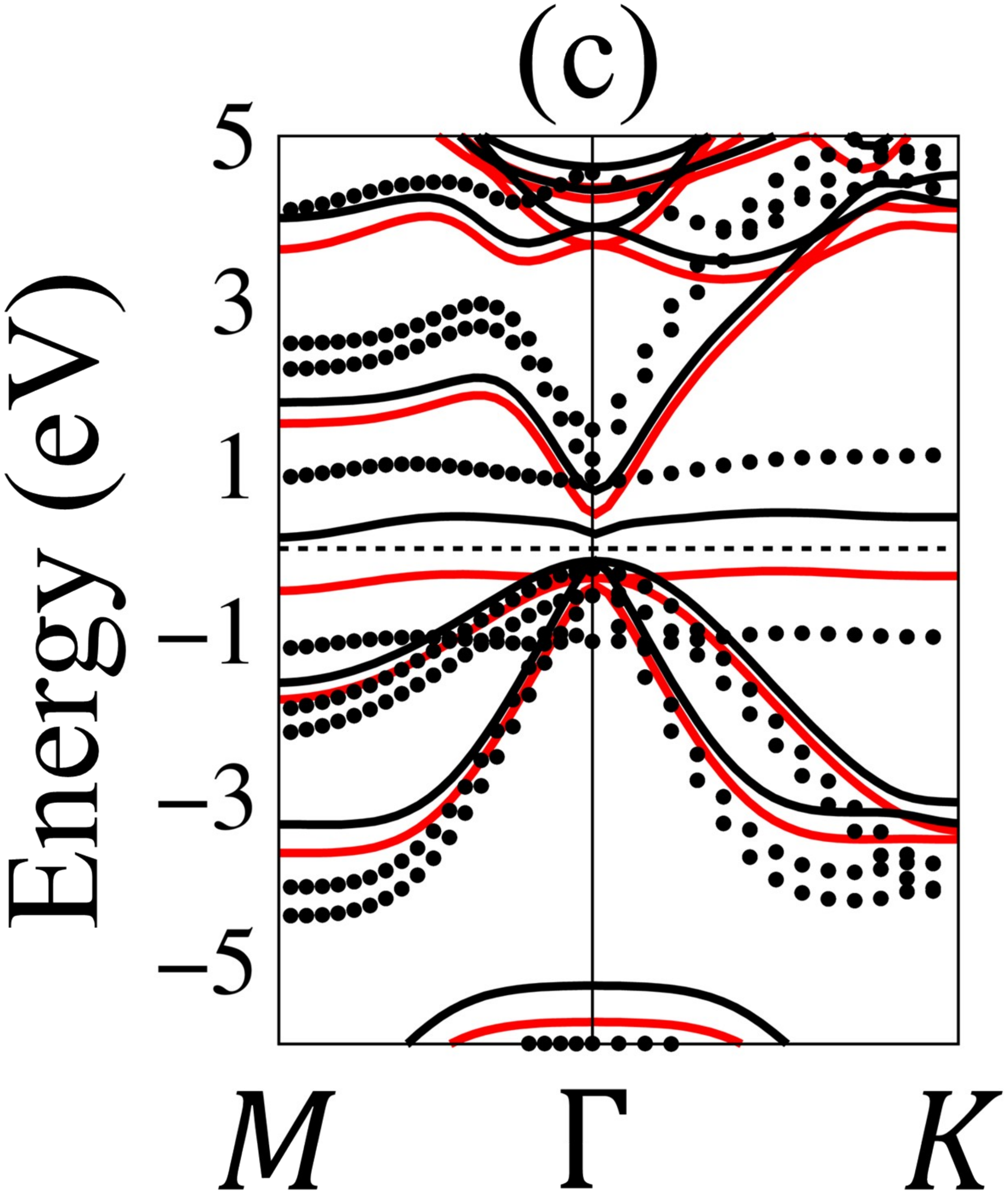

Figure S4: Band structure in DFT (Red solid-line: Spin-up, Black solid line: spin-down) and GW approximation (dot-line) for (a) graphone, (b) silicone, and (c) germanone, respectively. The zero indicates the Fermi level position.

(a) Graphone

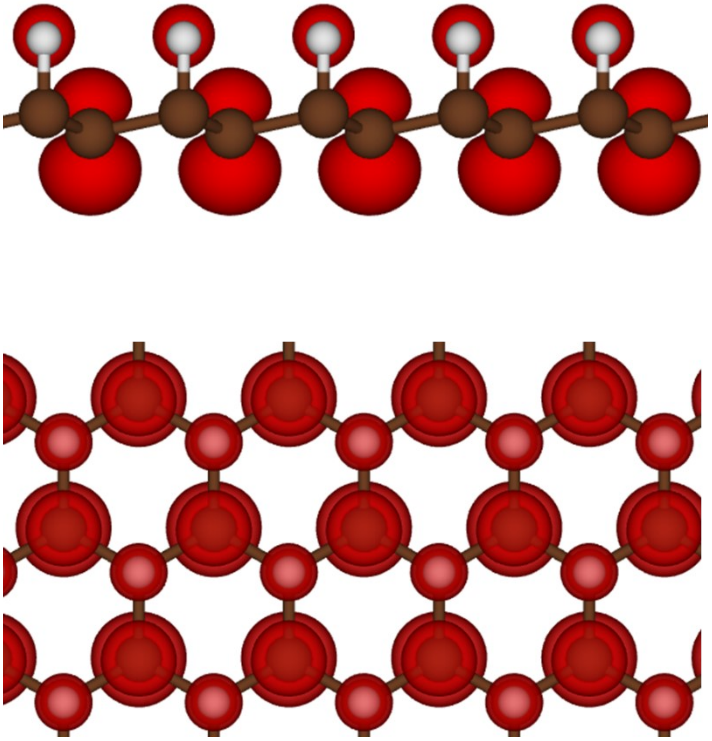

(b) Silicone

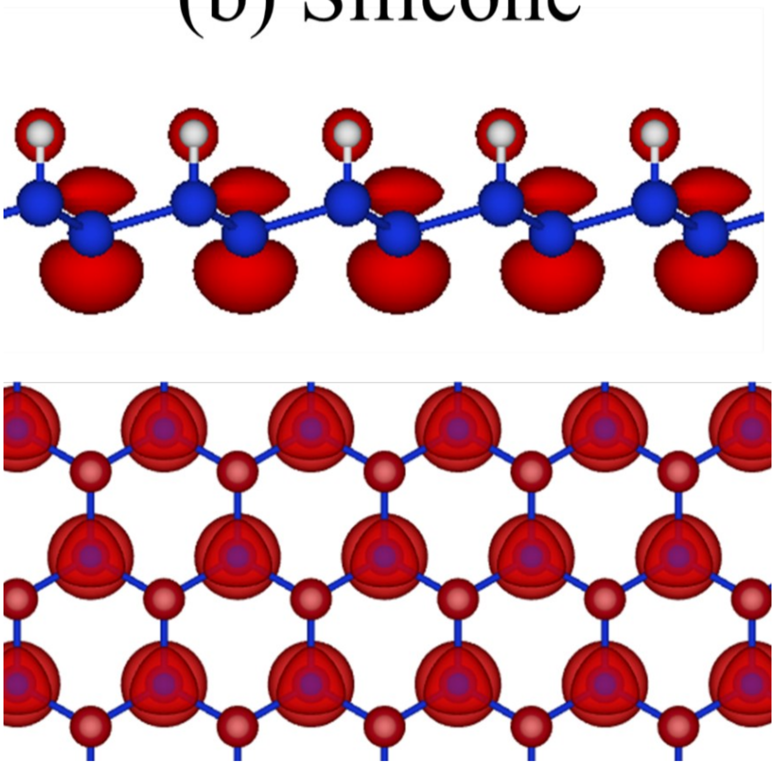

(c) Germanone

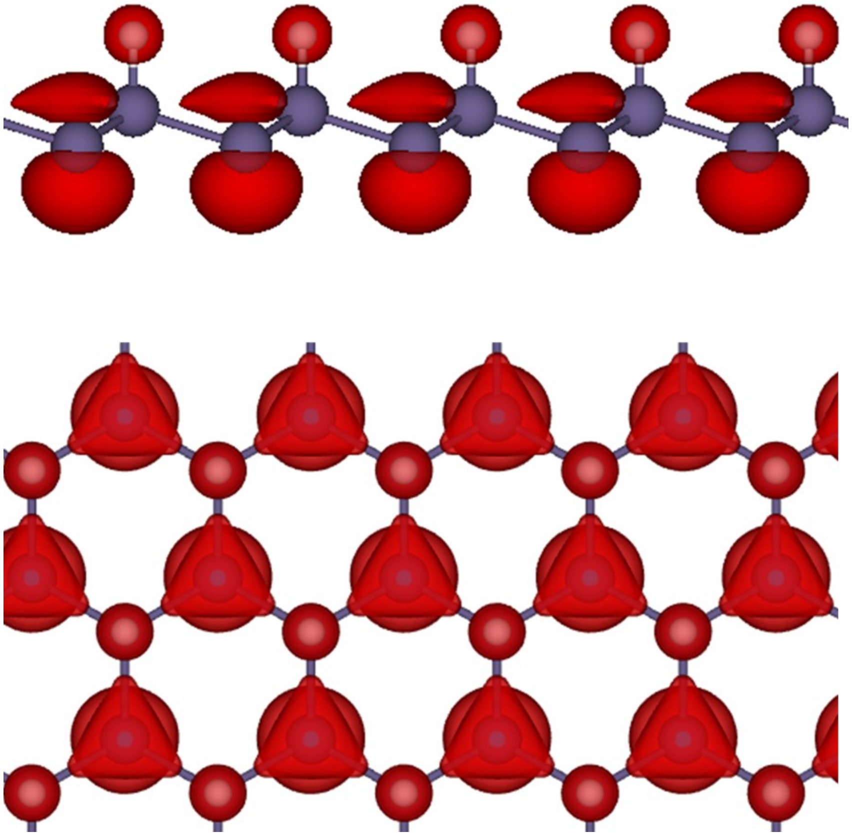

Figure S5. The spatial spin-density distribution of (a) graphone, (b) silicone, and (c) germanon.

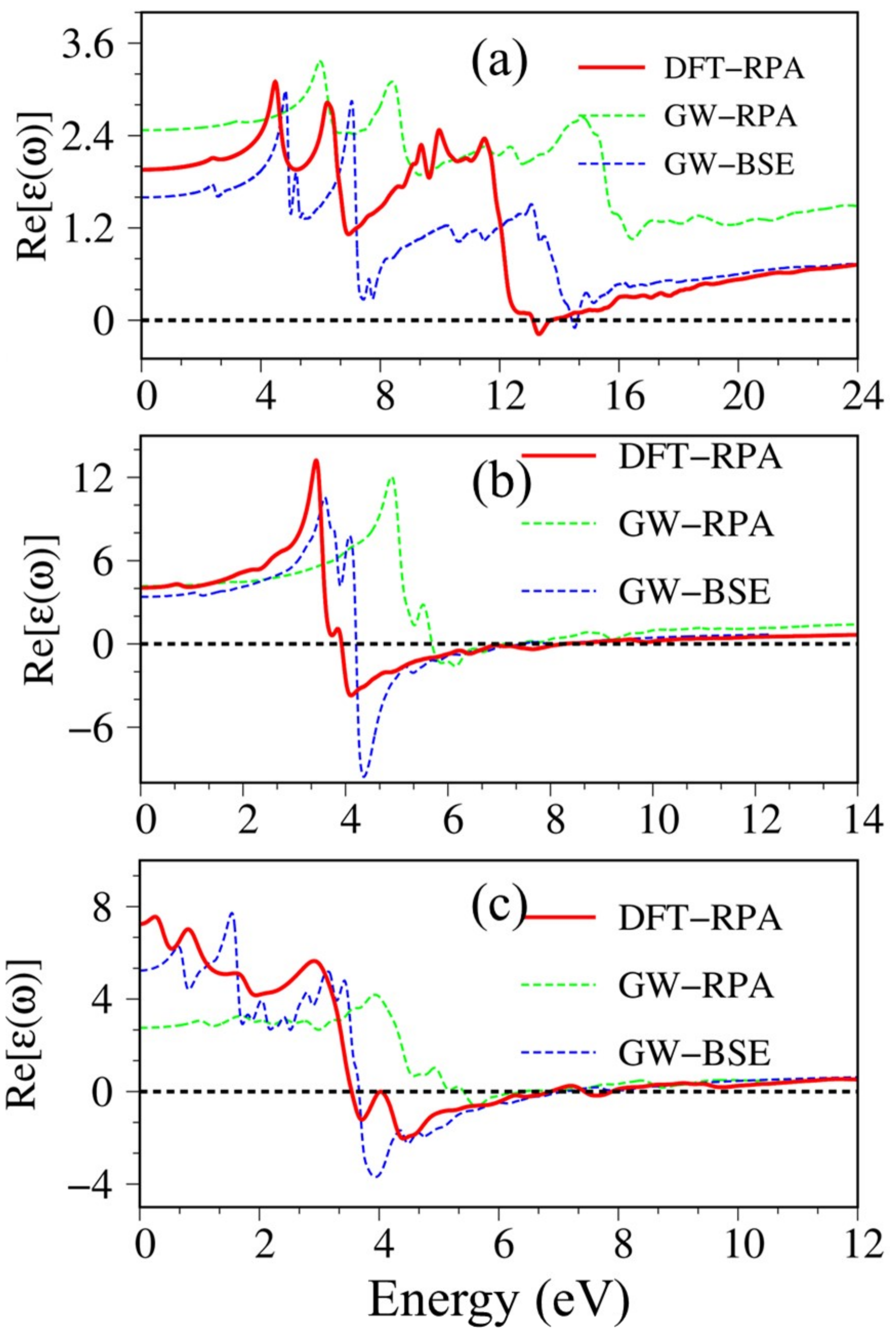

Figure S6: Real part of dielectric functions with different levels of theory of (a) graphone, (b) silicone, and (c) germanone. Red line: DFT-RPA; dashed-green line: GW-RPA; dashed-blue line: GW-BSE.

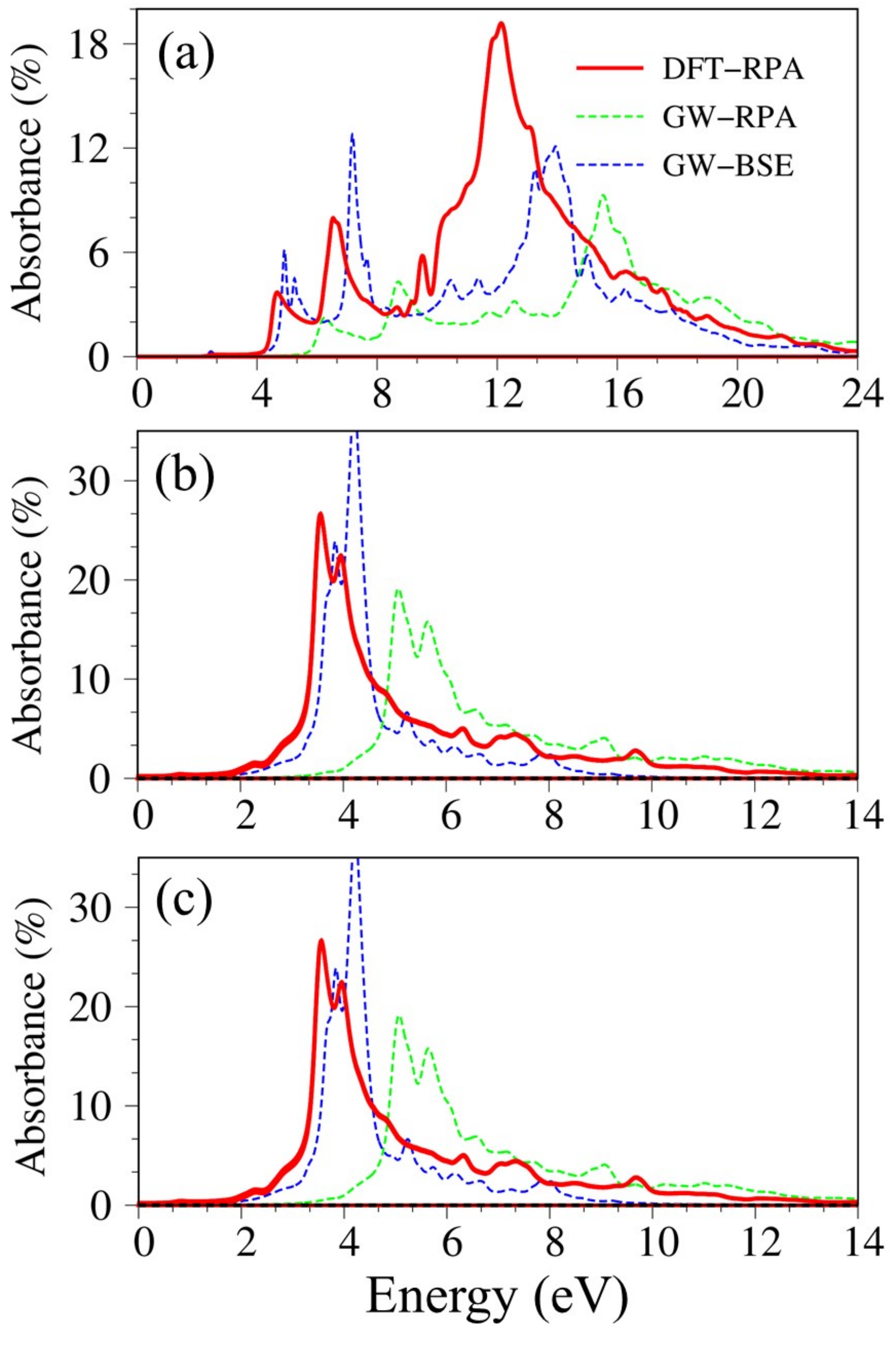

Figure S7: Absorbance coefficient with different levels of theory of (a) graphone, (b) silicone, and (c) germanone. Red line: DFT-RPA; dashed-green line: GW-RPA; dashed-blue line: GW-BSE.

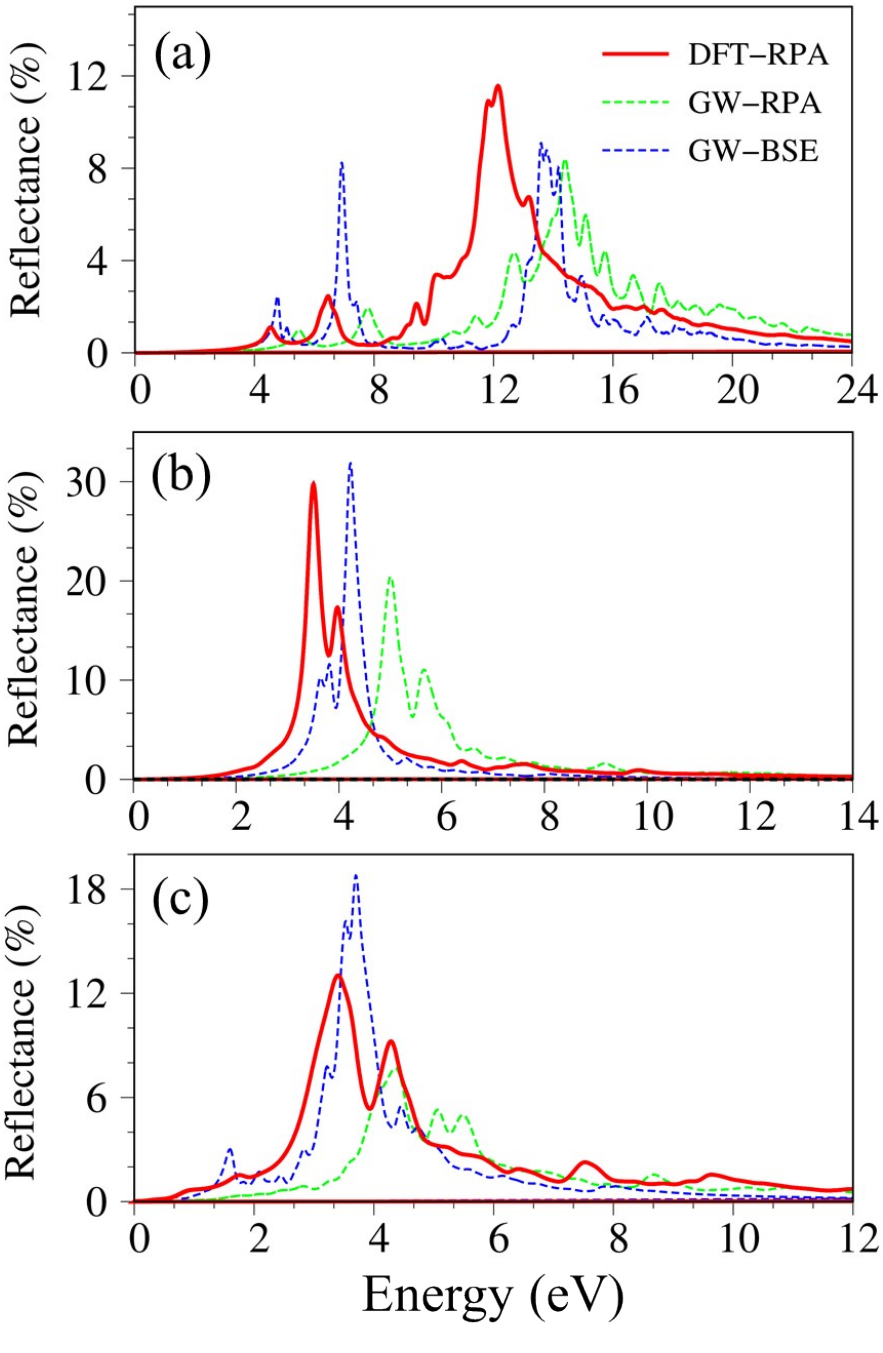

Figure S8: Reflectance coefficient with different levels of theory of (a) graphone, (b) silicone, and (c) germanone. Red line: DFT-RPA; dashed-green line: GW-RPA; dashed-blue line: GW-BSE.

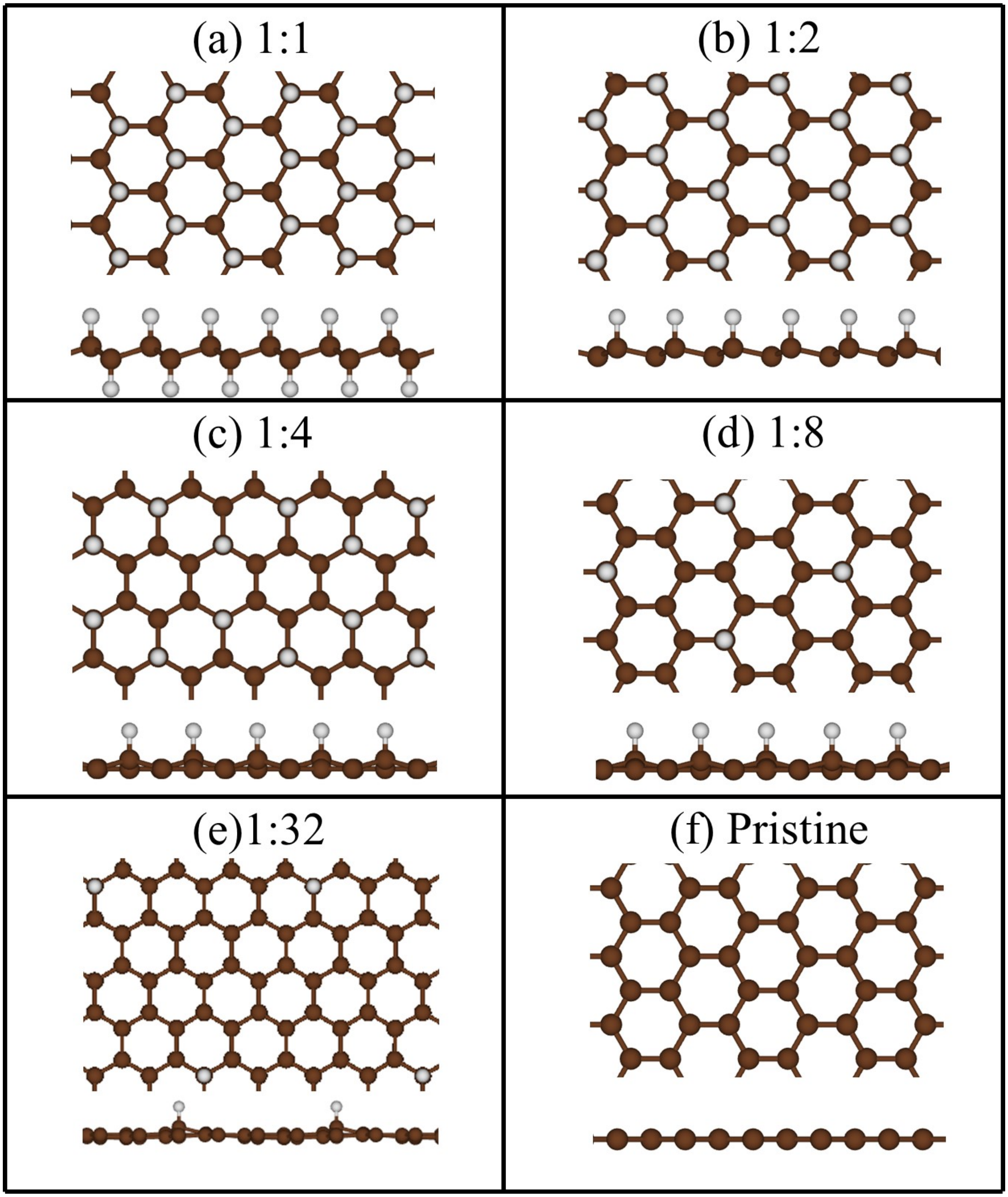

Figure S9: Geometric structures of hydrogenated graphene with top view and side view: (a) H:C 1:1, (b) H:C 1:2, (c) 1:4, (d) 1:8, (e) 1:32, and (f) Pristine graphene

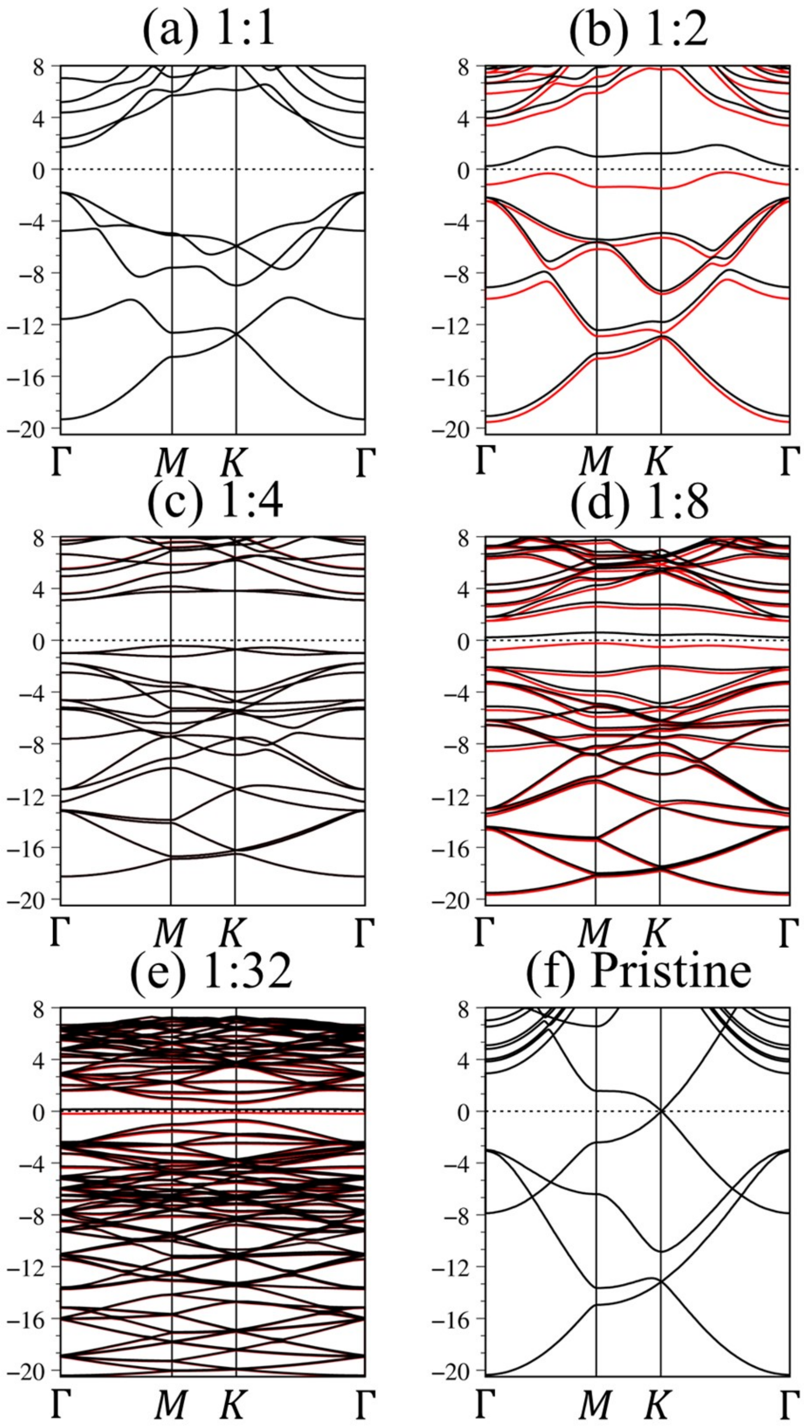

Figure S10: Electronic band structure of hydrogenated graphene along the high symmetry points: (a) H:C 1:1, (b) H:C 1:2, (c) 1:4, (d) 1:8, (e) 1:32, and (f) Pristine graphene. The red and black lines indicate the spin-up and spin-down states.

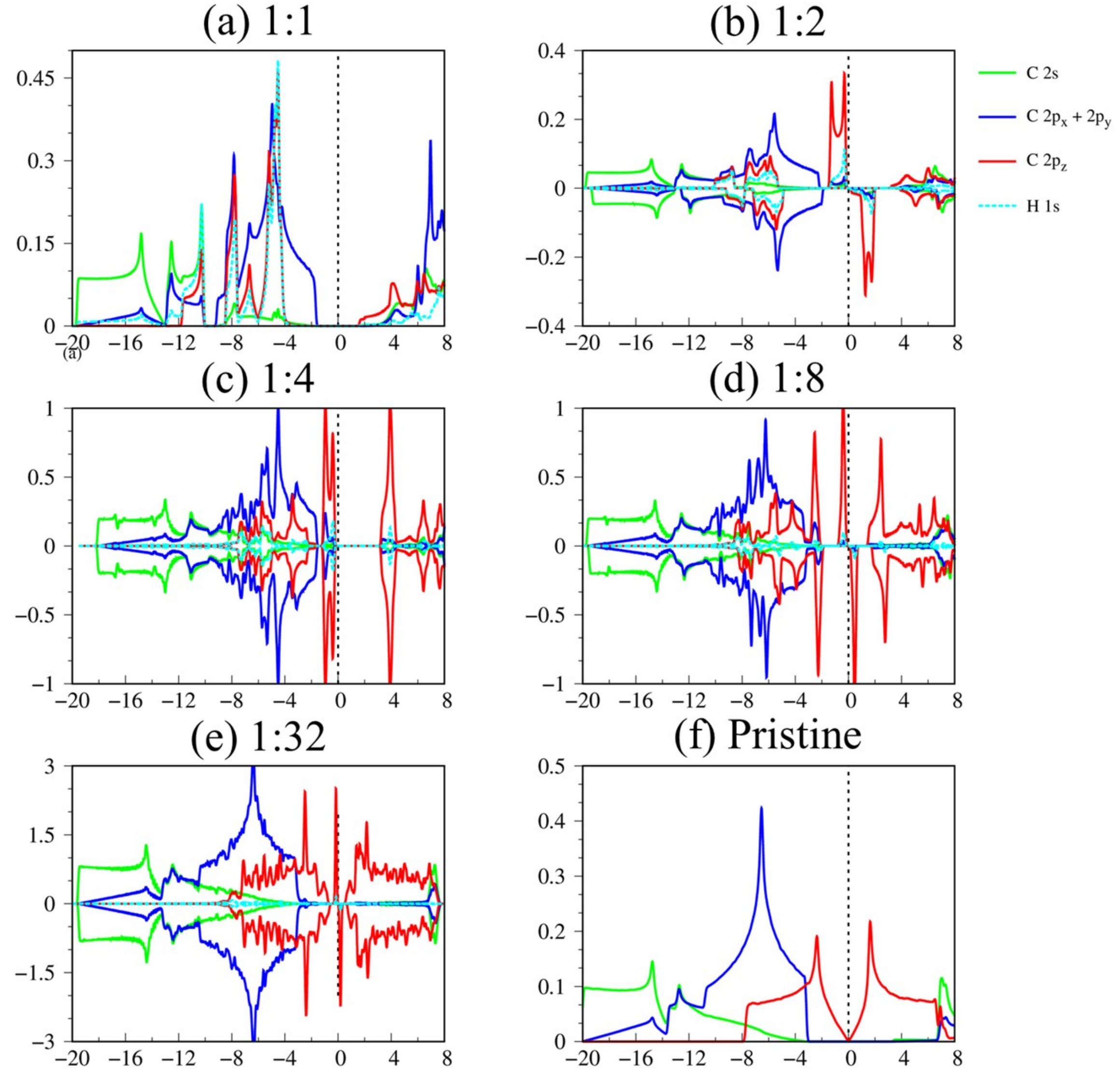

Figure S11: Orbital-projected density of states of hydrogenated graphene (a) H:C 1:1, (b) H:C 1:2, (c) 1:4, (d) 1:8, (e) 1:32, and (f) Pristine graphene.

Coordinate of graphene, silicene, germanene, graphone, silicone, and germanon

Graphene

```
pristine
   1.00000000000000
     2.4686369646199515    0.0000009389537138    0.0000000000000000
    -1.2343196619570387    2.1379016430259417    0.0000000000000000
     0.0000000000000000    0.0000000000000004   12.8258849135153099
   C
     2
Direct
  0.3333333279660948  0.1666666621132933  0.2894367685535784
  0.6666666406594146  0.8333333114549291  0.2894357690464204
```

Silicene

```
monolayer silicene
   1.00000000000000
     3.8678158403360636    0.0000000000000000    0.0000000000000000
    -1.9339079201680318    3.3496267748189927    0.0000000000000002
    -0.0000000000000001   -0.0000000000000001   15.0169449269651860
   Si
     2
Direct
  0.0000000000000000  0.0000000000000000  0.0834196557887736
  0.3333333129999971  0.6666666269999979  0.1133888542112302
```

Germanene

```
pristine
   1.00000000000000
     3.9678656514710546   -0.0000000002463613    0.0000000000041468
    -1.9839320492838008    3.4362388270763575    0.0000158718607410
     0.0000000000130468    0.0005824750623076   12.5012529623843971
   Ge
     2
Direct
  0.3333168728367397  0.1666337606734913  0.5095865621509077
  0.6666515661632602  0.8333033113265103  0.4578114558490896
```

Graphone

```
pristine
   1.00000000000000
     5.0588917732000001    0.0000000000000000    0.0000000000000000
    -2.5294464699999999    4.3811284537999997    0.0000000000000000
     0.0000000000000000    0.0000000000000000   13.1602191924999996
   C    H
     8     4
Direct
  0.1666666570000004  0.0833333280000019  0.4282482269999974
  0.1666666570000004  0.5833333129999971  0.4282482269999974
  0.6666666269999979  0.0833333280000019  0.4282482269999974
  0.6666666269999979  0.5833333129999971  0.4282482269999974
  0.3333333129999971  0.4166666570000004  0.4525303240000014
  0.3333333129999971  0.9166666269999979  0.4525303240000014
  0.8333333129999971  0.4166666570000004  0.4525303240000014
  0.8333333129999971  0.9166666269999979  0.4525303240000014
  0.3333333129999971  0.4166666570000004  0.5408812760000004
  0.3333333129999971  0.9166666269999979  0.5408812760000004
  0.8333333129999971  0.4166666570000004  0.5408812760000004
  0.8333333129999971  0.9166666269999979  0.5408812760000004
```

Silicone

```
monolayer
   1.00000000000000
     7.7979974746999998    0.0000000000000000    0.0000000000000000
    -3.8989996366000002    6.7532633925000001    0.0000000000000000
     0.0000000000000000    0.0000000000000000   13.2999334334999997
   Si   H
     8     4
Direct
  0.0000000000000000  0.0000000000000000  0.2857015430000018
  0.0000000000000000  0.5000000000000000  0.2857015430000018
  0.5000000000000000  0.0000000000000000  0.2857015430000018
  0.5000000000000000  0.5000000000000000  0.2857015430000018
  0.1666666570000004  0.3333333129999971  0.3363133670000025
  0.1666666570000004  0.8333333129999971  0.3363133670000025
  0.6666666269999979  0.3333333129999971  0.3363133670000025
  0.6666666269999979  0.8333333129999971  0.3363133670000025
  0.1666666570000004  0.3333333129999971  0.4503254890000008
  0.1666666570000004  0.8333333129999971  0.4503254890000008
  0.6666666269999979  0.3333333129999971  0.4503254890000008
  0.6666666269999979  0.8333333129999971  0.4503254890000008
```

Germanone

```
New structure
   1.00000000000000
     8.1914176941000001    0.0000000000000000    0.0000000000000000
    -4.0957097917000000    7.0939752706999997    0.0000000000000000
     0.0000000000000000    0.0000000000000000   13.7911281585999994
   Ge   H
     8     4
Direct
  0.1666664930000010  0.3333334920000013  0.2909573609999967
  0.1666664930000010  0.8333334920000013  0.2909573609999967
  0.6666665079999987  0.3333334920000013  0.2909573609999967
  0.6666665079999987  0.8333334920000013  0.2909573609999967
  0.3333334920000013  0.1666664930000010  0.2361388360000021
  0.3333334920000013  0.6666665079999987  0.2361388360000021
  0.8333334920000013  0.1666664930000010  0.2361388360000021
  0.8333334920000013  0.6666665079999987  0.2361388360000021
  0.1666664930000010  0.3333334920000013  0.4048893149999984
  0.1666664930000010  0.8333334920000013  0.4048893149999984
  0.6666665079999987  0.3333334920000013  0.4048893149999984
  0.6666665079999987  0.8333334920000013  0.4048893149999984
```

Hydrogenated graphene (1:1)

```
New structure
   1.00000000000000
     2.5502681732000001    0.0000000000000000    0.0000000000000000
    -1.2751340866000000    2.2085970245000000    0.0000000000000000
    -0.0005470133000000    0.0003158183000000   12.9999999847000005
   C    H
     2     2
Direct
  0.3334148530000007  0.6665851469999993  0.7207491400000023
  0.6667435170000005  0.3332564829999995  0.6847753520000026
  0.3334290390000021  0.6665709019999966  0.8060579899999993
  0.6667293309999991  0.3332706690000009  0.5994592309999973
```

Hydrogenated graphene (1:4)

```
pristine
   1.00000000000000
     4.9773260784138129    0.0000002159103783    0.0000000000000000
```

```
  -2.4886634262167533    4.3104906033982164    0.0000000000000000
   0.0000000000000000    0.0000000000000000   13.5950776043864821
 C    H
   8     2
Direct
 0.1624749174285908  0.0749498488133627  0.4338389754400851
 0.1624749173847709  0.5875250526152267  0.4338389754400851
 0.6666666269999979  0.0833333280000019  0.4606952719080478
 0.6750501061866367  0.5875250525714066  0.4338389754400851
 0.3333333129999971  0.4166666570000004  0.4606963038111156
 0.3249516797714893  0.9124758104076505  0.4338373110349096
 0.8375241295923446  0.4250482902285084  0.4338373110349096
 0.8375241296361644  0.9124758103638304  0.4338373110349096
 0.3333333129999971  0.4166666570000004  0.5430382639369301
 0.6666666269999979  0.0833333280000019  0.5430357340795754
```

Hydrogenated graphene (1:8)

```
pristine
   1.00000000000000
     4.9684880895131869    0.0000002796638523    0.0000000000000000
    -2.4842443755351065    4.3028367129496674    0.0000000000000000
     0.0000000000000000    0.0000000000000000   13.6434864975428418
   C    H
     8     1
Direct
  0.1633553057201660  0.0767106254080623  0.4367490225883435
  0.1633553056878953  0.5866446643121024  0.4367490225883435
  0.6666666269999979  0.0833333280000019  0.4397304983059590
  0.6732893295919303  0.5866446642798315  0.4367490225883435
  0.3333333129999971  0.4166666570000004  0.4616022035448679
  0.3307642066268385  0.9153820738195175  0.4360762449174927
  0.8346178661804775  0.4192357633731592  0.4360762449174927
  0.8346178661926684  0.9153820738073265  0.4360762449174927
  0.3333333129999971  0.4166666570000004  0.5441869756316459
```

Hydrogenated graphene (1:32)

```
pristine
   1.00000000000000
     9.9267526144682137    0.0000007425537069    0.0000000000000000
    -4.9633768089337709    8.5968196515472197    0.0000000000000000
     0.0000000000000000    0.0000000000000000   13.6716035359422055
   C    H
```

```
  32     1
Direct
  0.0812162988410487  0.0374326056766507  0.4467318732242301
  0.0812162988356087  0.2937836861643938  0.4467318732242301
  0.0830026062359705  0.5417573321072247  0.4337984339646480
  0.0830026062230261  0.7912453201146569  0.4337984339646480
  0.3334997867203222  0.0415001902796727  0.4439068649764361
  0.3375673713233435  0.2937836861589536  0.4467318732242301
  0.3334997867254167  0.5419996344457467  0.4439068649764361
  0.3327981737748119  0.7913991173872736  0.4416233319796740
  0.5836008826127264  0.0422018032251827  0.4416233319796740
  0.5837546798853437  0.2919973787769763  0.4337984339646480
  0.5837546798841955  0.5417573321213183  0.4337984339646480
  0.5836008826124675  0.7913991173875325  0.4416233319796740
  0.8330003655542532  0.0415001902745780  0.4439068649764361
  0.8332426678927745  0.2919973787640309  0.4337984339646480
  0.8333333129999971  0.5416666870000029  0.4295301572925330
  0.8332426678786817  0.7912453201158053  0.4337984339646480
  0.1666666570000004  0.2083333129999971  0.4726715690058509
  0.1654548384582946  0.4579016941058117  0.4415714125197474
  0.1668291804366451  0.7084145747182566  0.4351641705840036
  0.1654548384505829  0.9575531133444212  0.4415714125197474
  0.4174468566555770  0.2095451315494146  0.4415714125197474
  0.4174468566552472  0.4579016941138537  0.4415714125197474
  0.4162671222459470  0.7075342434847937  0.4429835079725963
  0.4162671222388437  0.9587328477611543  0.4429835079725963
  0.6665853652817384  0.2081707895633525  0.4351641705840036
  0.6668327447612262  0.4584163718887619  0.4309568775364101
  0.6665853652815984  0.7084145747183968  0.4351641705840036
  0.6674656965152010  0.9587328477540504  0.4429835079725963
  0.9170982458941830  0.2095451315417024  0.4415714125197474
  0.9165835681275341  0.4584163718724599  0.4309568775364101
  0.9165835681112334  0.7081671952387698  0.4309568775364101
  0.9170982458861413  0.9575531133447508  0.4415714125197474
  0.1666666570000004  0.2083333129999971  0.5548174079751246
```

References

[1] M. L. Cohen and S. G. Louie, *Fundamentals of condensed matter physics*. Cambridge University Press, 2016.

[2] G. Samsonidze, M. Jain, J. Deslippe, M. L. Cohen, and S. G. Louie, "Simple Approximate Physical Orbitals for G W Quasiparticle Calculations," *Physical Review Letters,* vol. 107, no. 18, p. 186404, 2011.

[3] M. P. Surh, S. G. Louie, and M. L. Cohen, "Quasiparticle energies for cubic BN, BP, and BAs," *Physical Review B,* vol. 43, no. 11, p. 9126, 1991.

[4] M. Fox, "Optical properties of solids," ed: American Association of Physics Teachers, 2002.

[5] R. Cutkosky, "Solutions of a Bethe-Salpeter equation," *Physical Review,* vol. 96, no. 4, p. 1135, 1954.

[6] S. Hirata and M. Head-Gordon, "Time-dependent density functional theory within the Tamm–Dancoff approximation," *Chemical Physics Letters,* vol. 314, no. 3-4, pp. 291-299, 1999.

[7] K. F. Mak, J. Shan, and T. F. Heinz, "Seeing Many-Body Effects in Single- and Few-Layer Graphene: Observation of Two-Dimensional Saddle-Point Excitons," *Physical Review Letters,* vol. 106, no. 4, p. 046401, 01/25/ 2011, doi: 10.1103/PhysRevLett.106.046401.
